# PUCHI represses early meristem formation in developing lateral roots of *Arabidopsis thaliana*

**DOI:** 10.1093/jxb/erac079

**Published:** 2022-02-28

**Authors:** Kevin Bellande, Duy-Chi Trinh, Anne-Alicia Gonzalez, Emeric Dubois, Anne-Sophie Petitot, Mikaël Lucas, Antony Champion, Pascal Gantet, Laurent Laplaze, Soazig Guyomarc’h

**Affiliations:** DIADE, Univ Montpellier, IRD, Montpellier, France; DIADE, Univ Montpellier, IRD, Montpellier, France; Univ Montpellier, CNRS, INSERM, Montpellier, France; Montpellier GenomiX, France Génomique, Montpellier, France; Univ Montpellier, CNRS, INSERM, Montpellier, France; Montpellier GenomiX, France Génomique, Montpellier, France; DIADE, Univ Montpellier, IRD, Montpellier, France; DIADE, Univ Montpellier, IRD, Montpellier, France; DIADE, Univ Montpellier, IRD, Montpellier, France; DIADE, Univ Montpellier, IRD, Montpellier, France; DIADE, Univ Montpellier, IRD, Montpellier, France; DIADE, Univ Montpellier, IRD, Montpellier, France; Michigan State University, USA

**Keywords:** Arabidopsis, auxin, cytokinin, lateral root development, meristem, organogenesis, patterning, PLETHORA, PUCHI

## Abstract

Lateral root organogenesis is a key process in the development of a plant’s root system and its adaptation to the environment. During lateral root formation, an early phase of cell proliferation first produces a four-cell-layered primordium, and only from this stage onwards is a root meristem-like structure, expressing root stem cell niche marker genes, being established in the developing organ. Previous studies reported that the gene regulatory network controlling lateral root formation is organized into two subnetworks whose mutual inhibition may contribute to organ patterning. *PUCHI* encodes an AP2/ERF transcription factor expressed early during lateral root primordium development and required for correct lateral root formation. To dissect the molecular events occurring during this early phase, we generated time-series transcriptomic datasets profiling lateral root development in *puchi-1* mutants and wild types. Transcriptomic and reporter analyses revealed that meristem-related genes were expressed ectopically at early stages of lateral root formation in *puchi-1* mutants. We conclude that, consistent with the inhibition of genetic modules contributing to lateral root development, PUCHI represses ectopic establishment of meristematic cell identities at early stages of organ development. These findings shed light on gene network properties that orchestrate correct timing and patterning during lateral root formation.

## Teaser

Consistent with the hypothesis that mutual inhibition between gene regulatory subnetworks contributes to lateral root formation, we show that *PUCHI*represses early meristematic gene expression in developing root primordia.

## Introduction

Dynamic and plastic development evolved in sessile organisms such as plants to adapt to changing environmental conditions (Morris *et al.*, 2017). Root branching, the production of new lateral roots (LRs) from existing roots, is key in elaborating root system architecture (Motte *et al.*, 2019). In the model plant *Arabidopsis thaliana*, LRs arise from pre-branch sites originating in the basal meristem close to the root apex (Xuan *et al.*, 2020). Primed pericycle cells at these pre-branch sites enter new rounds of cell divisions producing a lateral root primordium (LRP). The developing LRP, while growing through overlying cell layers, progressively organizes into a new root apical meristem (RAM) that ultimately emerges from the parent root and whose activity will determine the later growth of the LRs (Malamy *et al.*, 1997; Banda *et al.*, 2019). Remarkably, LRP development is a two-step process featuring an early, so-called morphogenetic phase (stages I–IV) during which cell proliferation generates a four-cell-layered, bulge-shaped LRP and a second, so-called meristematic phase (stages V–VIII) when the LRP further develops to acquire key RAM characteristics (Lucas *et al.*, 2013; Lavenus *et al.*, 2015; Voß *et al.*, 2015; Goh *et al.*, 2016). However, the mechanisms controlling the establishment of a new root meristem and especially the transition from the early phase of LRP development to the meristematic phase, when key root meristem genes start to be expressed in the centre of the developing LRP, remain unclear.

RAMs are proliferating tissues located at the tip of growing roots within which cell division and differentiation are regulated sequentially. Maintenance of a stem cell niche at the centre of root meristems ensures continuous production of cells and indeterminate organ growth (van den Berg *et al.*, 1995, 1997; Bennett and Scheres, 2010; Trinh *et al.*, 2018). Multiple regulatory components, including transcriptional regulators and hormones, mediate the regulation of cell identity and division activity in RAMs (Himanen *et al.*, 2002; Goh *et al.*, 2012, 2016; Porco *et al.*, 2016; Du and Scheres, 2017, 2018). In *A. thaliana*, networks of key transcription factors, including AP2-domain PLETHORA (PLT), GRAS-family transcription factors SHORT-ROOT (SHR) and SCARECROW (SCR), as well as auxin-dependent factors, have been shown to control the patterning of the RAM stem cell niche and specification of its organizing centre, known as the quiescent centre (QC) (Galinha *et al.*, 2007; Hofhuis *et al.*, 2013; Goh *et al.*, 2016; Du and Scheres, 2017; Shimotohno *et al.*, 2018). Many of these corresponding genes are also dynamically expressed during the process of LR formation (Tian *et al.*, 2014; Lavenus *et al.*, 2015; Goh *et al.*, 2016; Du and Scheres, 2017).

Several studies have shown that cell fate acquisition in developing LRPs is not dependent on cell lineage but relies on tissue-scale mechanisms and positional signals (Lucas *et al.*, 2013; von Wangenheim *et al.*, 2016). The progressive organization of the organ could thus result from emerging properties of a complex genetic network possibly integrating chemical and mechanical cues (Banda *et al.*, 2019). Two examples of such patterning mechanisms are the definition of inner and outer domains of the early LRP by the interplay of SHR and SCR expression domains, and the correct patterning of cell divisions at early stages and transition of the developing LRP to the meristematic phase by distinct sets of *PLT* genes (Goh *et al.*, 2016; Du and Scheres, 2017). These data highlight that gene regulatory events during early LRP development are necessary to transition to the second meristem organization phase.

Inference of the gene regulatory network (GRN) during LR formation suggested that two distinct genetic subcircuits operate and that their mutual inhibition could be instrumental in LRP developmental patterning (Lavenus *et al.*, 2015). The first one includes genes predominantly expressed in the whole primordium in the early stages, but only in its base in the later phase. The second one includes meristematic genes whose expression initiates during the transition from the morphogenetic phase to the meristematic phase in the tip of the developing primordium, where the new root meristem stem cell niche is established. Mutual inhibition between these two groups of genes may explain the bifurcation between cell identities in the central zone and the flanking domain of the developing LRP (Lavenus *et al.*, 2015). In addition, in complex genetic systems, mutual inhibition motifs associated with positive regulatory cascades can generate sequential waves in gene expression dynamics and control cell state switch over time (Alon, 2007).

PUCHI is an AP2/ERF-family transcription factor belonging to the first GRN subcircuit and whose loss of function or perturbation in expression kinetics impairs LRP development (Hirota *et al.*, 2007; Kang *et al.*, 2013; Trinh *et al.*, 2019; Toyokura *et al.*, 2019; Goh *et al.*, 2019). GRN inference and experimental validation previously revealed that PUCHI acted as a master regulator of very long chain fatty acid (VLCFA) biosynthesis during the first phase of LRP development (Trinh *et al.*, 2019). However, the role played by PUCHI-dependent genetic pathways in LRP development remains elusive.

Here, we show that the AP2/ERF transcription factor PUCHI, which is predominantly expressed at early stages of LR development, controls the correct timing and pattern of expression of transcription factors as well as the distribution of hormonal signals that orchestrate meristem formation during late LR organogenesis. Root meristem-related genes are ectopically expressed in inner cell layers of early-phase LRPs in a *puchi-1* mutant background. Thus, these results support the hypothesis that the inhibition of gene regulatory modules participates in LRP functional patterning, and show that in the absence of PUCHI, the LR GRN yields premature expression of meristematic genes in inner cells of young LRPs.

## Materials and methods

### Plant materials, constructs, and growth conditions

*Arabidopsis thaliana* ecotype Columbia-0 (Col-0) plants were used for all experiments and as the background of transgenic lines in this study. The *A. thaliana* seeds were surface-sterilized and sown on half-strength Murashige and Skoog (1/2 MS) solid medium with 0.7% (w/v) plant agar supplemented with B5 vitamins. Plates were kept at 4 °C for 2 d and then placed in long-day conditions (16 h light/8 h dark cycle) in a vertical position. The *puchi-1* mutant line has previously been described (Hirota *et al.*, 2007). The *promPLT:PLT-YFP* lines were reported in Galinha *et al.* (2007) and Hofhuis *et al.* (2013). The *DR5::GFP* synthetic auxin response reporter was described in Friml *et al.* (2003), the synthetic cytokinin response reporter *promTCSn::GFP* was described in Zürcher *et al.* (2013), and the organizing centre markers *QC25::CFP* and *promWOX5::nls:GFP* were described in ten Hove *et al.* (2010) and Goh *et al.* (2016) respectively.

### RNAseq

Col-0 wild-type (WT) and *puchi-1* mutant seeds were surface-sterilized and sown on 1/2 MS solid medium containing 0.7% (w/v) plant agar supplemented with B5 vitamins. Plates were kept at 4 °C for 2 d and then placed in continuous light conditions in a vertical position. Gravistimulation was performed on 4-day-old seedlings, and root bends were sampled at 12, 18, 24, 30, and 36 h after gravistimulation. Three biological replicates (for both Col-0 and *puchi-1*) were used for the RNAseq experiment. For each replicate, root bends of >400 seedlings were dissected under a binocular microscope and frozen in liquid nitrogen immediately on harvesting. Approximately 400 mature root segments located between the bend and the shoot were harvested in 4-day-old seedlings at 12 h after gravistimulation to be used as a reference of root tissues devoid of developing LRPs (termed ‘No LR’ in the dataset). Total RNA was extracted using a Qiagen RNeasy plant mini kit with an on-column DNase treatment following the manufacturer’s recommendations (RNase-free DNase Set, Qiagen, Crawley, UK). RNA samples were quantified using a Nanodrop ND100 spectrophotometer (Nanodrop, Wilimington, DE, USA), and RNA purity and integrity were evaluated using High-Resolution Automated Electrophoresis 2100 Bioanalyzer system from Agilent Technologies (https://www.agilent.com/en/product/automated-electrophoresis).

RNAseq analyses was performed by the MGX-Montpellier GenomiX platform (https://www.mgx.cnrs.fr/). cDNA libraries were constructed using a Stranded mRNA Prep Ligation kit (Illumina, San Diego, CA, USA) according to the manufacturer’s instructions. Briefly, poly(A) RNAs were purified using oligo-d(T) magnetic beads from 310 ng of total RNA. Poly(A) RNAs were fragmented and underwent reverse transcription using random hexamers. During the second-strand generation step, dUTP substituted dTTP to prevent the second strand being used as a matrix during the final PCR amplification. Double-stranded cDNAs were adenylated at their 3ʹ ends and ligated to Illumina’s universal anchors. Ligated cDNAs were amplified following 12 PCR cycles. During this PCR, the libraries were indexed, and the adapter’s sequences were completed to be compatible with the cluster generation step. PCR products were purified using AMPure XP Beads (Beckman Coulter Genomics, Brea, CA, USA). Libraries were validated using a High Sensitivity NGS kit on a Fragment Analyzer (Agilent Technologies, Santa Clara, CA, USA) and quantified using a KAPA Library quantification kit (Roche, Basel, Switzerland).

For library sequencing, 36 libraries were pooled in equimolar amounts. The balance between all samples of the pool was assessed by sequencing on a Miniseq (Illumina) using a 300 cycle Mid Output Reagent Cartridge. The pool was then sequenced on a Novaseq 6000 (Illumina USA) S1 flow cell in paired-end 2*100 nt mode according to the manufacturer’s instructions. This sequencing produced between 36 and 44 million passed filter clusters per library.

For sequencing quality control, image analyses and base calling were performed using the NovaSeq Control Software and Real-Time Analysis component (Illumina). Demultiplexing and trimming were performed using Illumina’s conversion software (bcl2fastq 2.20). The quality of the raw data was assessed using FastQC from the Babraham Institute and the Illumina software SAV (Sequencing Analysis Viewer). FastqScreen was used to estimate the potential level of contamination.

For alignment and statistical analysis, a splice junction mapper, TopHat 2.1.1 (Kim *et al.*, 2013) (using Bowtie 2.3.5.1; Langmead *et al.*, 2009), was used to align the RNAseq reads to the *A. thaliana* genome (NCBI TAIR10.1) with a set of gene model annotations (gff file downloaded from the NCBI on 13 November 2020). Final read alignments with more than six mismatches were discarded. Samtools (v1.9) was used to sort the alignment files. Then, gene counting was performed using Featurecounts 2.0.0 (Liao *et al.*, 2014). As the data are from a strand-specific assay, the reads needed to be mapped to the opposite strand of the gene (-s 2 option). Before statistical analysis, genes with fewer than 15 reads (cumulating all the analysed samples) were filtered out.

Differentially expressed genes (DEGs) were identified using the Bioconductor (Gentleman *et al.*, 2004) package (http://www.bioconductor.org) DESeq2 1.26.0 (Love *et al.*, 2014) (R version 3.6.1). Data were normalized using the DESeq2 normalization method. Genes with an adjusted *P*-value of <5% (according to the false discovery rate method of Benjamini and Hochberg) were declared differentially expressed. DEGs between Col-0 and *puchi-1* were then investigated for each time point. The DEGs with a log2 fold change (FC) >1 or log2FC <–1 and a DESeq2 package Wald test (*P*-value: *P<0.05; **P<0.02; ***P<0.01) were selected for the Gene Ontology (GO) analysis. GO biological process enrichment analyses of the list of either up- or down-regulated DEGs in *puchi-1* were performed using a PANTHER Overrepresentation assay (http://pantherdb.org) and Fisher’s test followed by a Bonferroni correction (*P<0.05) (Thomas *et al.*, 2003; Mi *et al.*, 2013). Only the individual elementary annotations of the GO biological process are shown.

### RT–qPCR analysis

For quantitative reverse transcription–PCR (RT–qPCR) analyses, plant material was collected following the lateral root induction system described in Himanen *et al.* (2002). Briefly, square Petri dishes containing 1/2 MS solid medium, 0.7% (w/v) plant agar supplemented with B5 vitamins, and containing *N*-1-naphthylphthalamic acid (NPA) and/or 1-naphthalene acetic acid (NAA) were used. Col-0 and *puchi-1* seeds were germinated and grown for 72 h on 5 µM NPA before transfer to 5 µM NPA-containing (control) or 10 µM NAA-containing (LR induction) medium. Three biological replicates of root segments without the RAM were harvested after 24 h of treatment and immediately frozen in liquid nitrogen for each condition. Total RNA was extracted using a Qiagen RNeasy plant mini kit with an on-column DNase treatment following the manufacturer’s recommended protocol (RNase-free DNase Set, Qiagen). Quantification, purity, and contamination assessment of the RNA samples was carried out using gel electrophoresis and a Nanodrop ND100 spectrophotometer. cDNAs generated from the three biological replicates were synthesized following the manufacturer’s recommended protocol (Omniscript RT Kit, Qiagen): 1000 ng of RNA in RNase-free water were heated 10 min at 70 °C, and added to the manufacturer’s mix (up to 20 µl) for 1 h at 37 °C. Generated cDNAs diluted 1/10 were added to the Brilliant III Ultra-Fast SYBR® Green QPCR Master Mix with Low ROX reaction mix and used to perform qPCR analysis as recommended by the manufacturer (Qiagen). All qPCRs were carried out and analysed using the Roche® LC480 Lightcycler system and software (qPCR analysis program, Cq determination, curves calibration). Complete thermocycling parameters were as follow: 95 °C, 3 min 15 s; 95 °C, 20 s; 60 °C, 20 s ×40 cycles; 95 °C, 1 min; 60 °C, 30 s; 95 °C, 30 s. Normalization was achieved with the tubulin *TUB3* gene (AT5G62700) and the *UBQ5* gene (AT3G62250) (Lavenus *et al.*, 2015; Trinh *et al.*, 2019). The calibrator cDNA for relative quantification of the effect of each treatment was the control WT treated with NPA. Data are represented as the mean ±SEM. Significant differences between NAA-treated samples were determined using a Student’s *t*-test (*P<0.05; **P<0.02; ***P<0.01). The primers used for RT–qPCR analysis were designed to generate amplicon lengths of 75–110 bases. Primer efficiency and specificity were confirmed by qPCR. Primer sequences were as follows: AT3G20840, PLT1-F CAACCCTTTTCAAACACAAGAGT and PLT1-R TTGGAACCTCTCCTCCTTCA; AT1G51190, PLT2-F AGGAAAGGAAGACAAGTCTACTTAGG and PLT2-R AGAGGGACCCCAATATTTAAGTG; AT5G10510, PLT3-F GATCTTTACCTTGGAACCTTTGC and PLT3-R GCTGCTATGTCATACGCTTCA; AT5G17430, PLT4/BBM-F GAGACAATAATAGTCACTCCCGAGAT and PLT4/BBM-R TTTGTTCGTTATTGTTAATGTTATTGC; AT5G57390, PLT5-F CTCCATGTACAGAGGCGTCA and PLT5-R GCAGCTTCCTCTTGAGTGCTA; AT5G65510, PLT7-F AACAGCTGTAGGAGGGAAGGT and PLT7-R TCTATCTTCCTTGTCATATCCACCTA; TUB3-F TGCATTGGTACACAGGTGAGGGAA and TUB3-R AGCCGTTGCATCTTGGTATTGCTG; and UBQ5-F CGATGGATCTGGAAAGGTTC and UBQ5-R AGCTCCACAGGTTGCGTTAG.

### Confocal microscopy, image processing, and analysis

Confocal microscopy images were obtained with a Leica SP8 confocal system using a ×40 HCX corr Plan Apochromat CS 1.1 NA water objective. Samples were mounted in water or in 15 µM propidium iodide for 1–2 h or in 15 µM propidium iodide supplemented with 0.004% Triton X-100 for 20–40 min as previously described (Du and Scheres, 2017). All combinatorial fluorescence analyses were run as sequential scans. The following excitation/emission settings were used to obtain specific fluorescence signals: for propidium iodide, 488/600–620 nm; for yellow fluorescent protein (YFP), 514/520–550 nm; for cyan fluorescent protein (CFP), 458/475–505 nm; and for enhanced green fluorescent protein (EGFP), 488/500–550 nm. All post-acquisition image such as channel merging was performed using Fiji (https://imagej.net/Fiji) (Schindelin *et al.*, 2012).

### Database mining for PUCHI target genes

Genes showing differential expression (fold change ≥1.5, *P*-value ≤0.05 as determined by Welch two sample *t*-test) in auxin-treated fusion protein PUCHI–GR (glucorticoid receptor) roots upon dexamethasone treatment and in the presence of cycloheximide were retrieved from Trinh *et al.* (2019). Genes associated with genome sequences bound by the transcription factor PUCHI *in vitro* (ampDAP, FRiP ≥5%) were retrieved from the Arabidopsis DAP-seq database published at http://neomorph.salk.edu/dev/pages/shhuang/dap_web/pages/index.php (O’Malley *et al.*, 2016). List contents were compared using the ‘Calculate and draw custom Venn diagrams’ system from VIB/ UGent Bioinformatics & Systems (https://www.vandepeerlab.org/?q=tools/venn-diagrams) to select genes that had been detected by at least two of the three methods: RNAseq profiling of *puchi-1* roots, DAP-seq using PUCHI as a bait, or inducible complementation of PUCHI–GR *puchi-1* roots. Aliases and description summaries were retrieved for each gene from Thalemine (https://bar.utoronto.ca/thalemine/begin.do). Over-representation of GO terms among the 150 genes that are common between two datasets was assayed using the PANTHER algorithm (http://pantherdb.org) and Fisher’s test followed by a Bonferroni correction (*P<0.05) (Thomas *et al.*, 2003; Mi *et al.*, 2013). Among the list of those 150 genes, transcription factors and genes annotated as related to auxin, cytokinin, VLCFA, LR development, or meristem regulation were selected for closer inspection (see Supplementary Table S1 for detailed lists).

## Results

### Loss of PUCHI function alters sequential expression patterns of *PLETHORA* genes in developing lateral root primordia

**Fig. 1.**
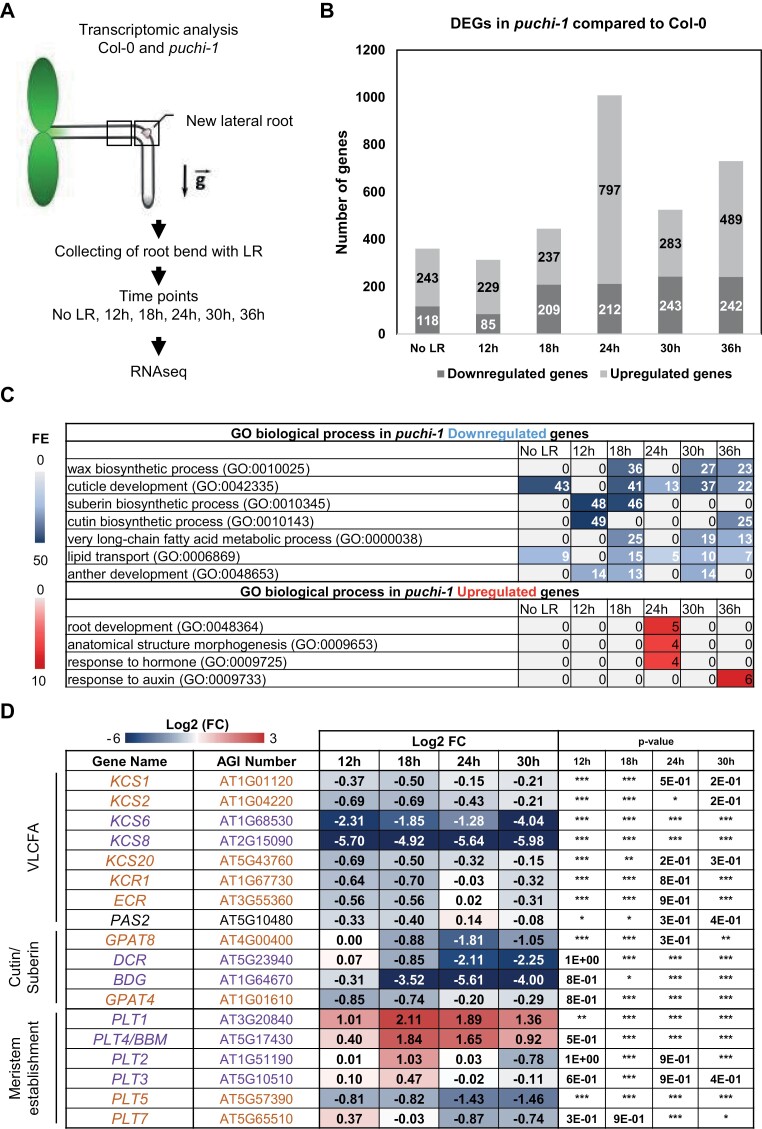
Time-course transcriptomic analysis of LRP formation in Col-0 and *puchi-1* after root gravistimulation. (A) Schematic diagram of the experimental procedure for the RNAseq analysis. (B) Number of differentially expressed genes (DEGs) in *puchi-1* compared with Col-0 at No LR, 12, 18, 24, 30, and 36 h after gravistimulation. Genes with a fold change (FC) of log2FC >1 or log2FC <–1 with *P<0.05 were selected. (C) Distribution of elementary Gene Ontology (GO) terms among either up-regulated or down-regulated genes in *puchi* was analysed using a PANTHER over-representation assay and Fisher test followed by a Bonferroni correction (*P<0.05). The heat map shows the GO term fold enrichment (FE) of DEGs for each time point. (D) Heat map of selected gene patterns up- and down-regulated in *puchi-1* compared with the Col-0 background during the formation of LRPs. Log2FC of expression in *puchi-1* compared with the WT is given. Statistical analysis on three independent RNAseq replicates was performed using the DESeq2 package and Wald test: *P<0.05; **P<0.02; ***P<0.01. The colour code for the heatmaps in (C) and (D) is red for up-regulated genes and blue for down-regulated genes. The colour code for gene names in (D) indicates the module classification (orange, module1, morphogenetic phase; and purple, module 2, meristematic phase) as described in Lavenus *et al.* (2015).

To assess the role played by PUCHI in the genetic regulation of LRP development and identify its targets, either direct or indirect, during this organogenesis process, we performed a time-series transcriptomic analysis following LR induction by gravistimulation in Col-0 WT and *puchi-1* mutant plants (Fig. 1A). Root segments were sampled at 12 hours post-gravistimulation (hpg; stage I), 18 hpg (predominantly stage II in Col-0 and *puchi-1*), 24 hpg (stages II–IV in Col-0, stages II–III in *puchi-1*), 30 hpg (stages III–V in Col-0, stages II–IV in *puchi-1*), and 36 hpg (stages III–VI in Col-0, stages II–V in *puchi-1*), which correspond approximately to LR initiation, the mid and late morphogenetic phases, and the early meristematic phase in a Col-0 background in our experimental conditions (Supplementary Fig. S1A–C) (Goh *et al.*, 2016; Trinh *et al.*, 2019). A root segment was sampled directly above the root bend in both genotypes at 12 hpg to be used as ‘No LR’ reference material. RNAseq profiling of these samples was performed, and DEGs between successive time points or between the two genotypes at a single time point were identified (log2FC >1 or log2FC < –1, *P<0.05; see the Materials and methods).

A total of 5791 genes in the WT and 9571 genes in the *puchi-1* mutant background were shown to be differentially expressed during the LRP developmental time course. Noticeably, comparison of WT and *puchi-1* reference ‘No LR’ root segments revealed 361 genes differentially expressed between the two genotypes (Fig. 1B). In contrast, comparison of *puchi-1* with WT LRP transcriptomic datasets at 12, 18, 24, 30, and 36 hpg yielded a minimum of 314 up to a maximum of 1009 DEGs, with the highest divergence between the two genotypes observed at 24 and 36 hpg (Fig. 1B). Because we were first interested in uncovering the role of PUCHI in the process of LR organogenesis without necessarily distinguishing the primary molecular targets of this transcription factor from the secondary regulatory events influencing LRP formation, we examined all the genes whose expression dynamics during LRP formation was shown to be dependent on PUCHI function, later termed PUCHI-dependent genes.

We analysed GO enrichment among DEGs focusing specifically on genes whose expression is either up-regulated or down-regulated in *puchi-1* as compared with the WT. Consistent with previous studies, this analysis revealed that PUCHI-dependent genes are highly enriched in lipid metabolism-related genes (*P*-value <0.001), and confirmed that PUCHI positively influences the expression of multiple VLCFA, cutin, and suberin biosynthetic genes during LRP formation (Fig. 1C, D; Supplementary Figs S2, S3) (Trinh *et al.*, 2019). In addition, transcriptomic profiling in *puchi-1* and the WT revealed other biological processes dependent on PUCHI. Interestingly, root development-related genes were over-represented in the *puchi-1* RNAseq dataset as compared with the WT (*P<0.05 when comparing *puchi-1* and the WT at 24 h). These include *PLT1*, *PLT2*, and *PLT4/BBM* genes, which are well known regulators of RAM establishment and maintenance (Fig. 1D) (Aida *et al.*, 2004). Other genes whose function can be related to root meristem organization or activity (e.g. predominantly expressed in the QC; Nawy *et al.*, 2005) displayed altered expression dynamics during LRP development in the *puchi-1* background (Supplementary Fig. S3). In particular our RNAseq analysis shows robust overexpression of *PLT1* and *PLT4* genes in *puchi-1* root segments throughout the four time points of our dataset and especially at 18 h and 24 h, which correspond to root segments harbouring young LRPs from stage II to stage III—before the transition to the meristematic phase (Fig. 1D; Supplementary Fig. S1). Conversely other regulators of the PLETHORA family, *PLT5* and *PLT7*, were down-regulated in *puchi-1* root segments as compared with the WT (Fig. 1D). These latter results are consistent with previously published LR GRN prediction analyses and confirm that both *PLT5* and *PLT7* are positively regulated downstream of *PUCHI* in the early morphogenetic phase (Lavenus *et al.*, 2015). Underexpression of *PLT5* and *PLT7* was confirmed by independent RT–qPCR analyses in *puchi-1* roots compared with the WT 24 h after LR induction by auxin (which corresponds to stages II–III LRPs in Col-0 and stages I–III LRPs in *puchi-1*) (Himanen *et al.*, 2002) (Supplementary Fig. S4A).

We took advantage of published *in vitro* and *in vivo* datasets to compare identified DEGs with previously reported PUCHI-dependent gene expression or PUCHI genomic binding sites. First, a list of 13 putative direct target genes of PUCHI were previously identified from the transcriptomic response of the roots of PUCHI–GR seedlings upon dexamethasone treatment and in the presence of cycloheximide, an inhibitor of protein synthesis (Trinh *et al.*, 2019). Second, a total of 2412 putative PUCHI-binding sites have been identified in the genome using DAP-seq technology (O’Malley *et al.*, 2016). Comparison of those lists with the list of PUCHI-dependent genes identified by RNAseq profiling of branching root segments (this work) yielded one single gene, namely *CYTOKININ RESPONSE FACTOR 1* (*CRF1*), that is retrieved by these three investigation methods, and a total of 150 genes identified by at least two of these three experimental strategies (Supplementary Fig. S5A; Supplementary Table S1). Because DAP-seq profiles genomic sequences for which the PUCHI transcription factor displays affinity *in vitro*, and because the PUCHI–GR complementation assay was performed in the presence of cycloheximide, *CRF1* is most probably a direct target of PUCHI that induces its expression during LRP formation (Supplementary Figs S3, S6E). Interestingly, this gene encodes a component of the cytokinin signalling pathway that modulates a wide range of developmental processes (Raines *et al.*, 2016) (Supplementary Fig. S5). Next, a GO analysis revealed that genes associated with responses to stresses and to chemical stimuli are over-represented among the 150 genes shared by at least two of these three experimental datasets (Supplementary Table S1). Transcription factor genes and genes associated with auxin and cytokinin homeostasis and signalling pathways, LR development, meristem regulation, and VLCFA biosynthesis are also on this list (Supplementary Fig. S5B; Supplementary Table S1). Remarkably, *PLT5*, whose expression dynamics in branching root segments are dependent on PUCHI (this work), was associated with PUCHI-binding sites identified by DAP-seq analysis. Thus, altogether these results suggest that the early expressed LRP regulator PLT5 may also be a direct target gene of PUCHI during LR development.

LRP development progression in a *puchi-1* mutant background has previously been shown to be delayed (Hirota *et al.*, 2007; Trinh *et al.*, 2019). To assess the role played by this developmental delay in the transcriptomic gap observed between *puchi-1* and WT LRPs, we analysed the expression profiles of selected genes whose time-dependent expression dynamics during LRP development have previously been documented (Lavenus *et al.*, 2015; Nagata *et al.*, 2021). In most cases, observed changes in gene expression kinetics between both genotypes were not consistent simply with shifted developmental time courses (Supplementary Figs S6, S7). Taken together, our data confirm that PUCHI activity is required for correct progression of LRP development and suggest that this transcription factor induces the expression of *CRF1*, *PLT5*, and *PLT7* genes, and represses the expression of several meristem-specific genes during the LRP early morphogenetic phase.

### Meristematic *PLT* genes are induced prematurely in *puchi-1* LRPs

**Fig. 2.**
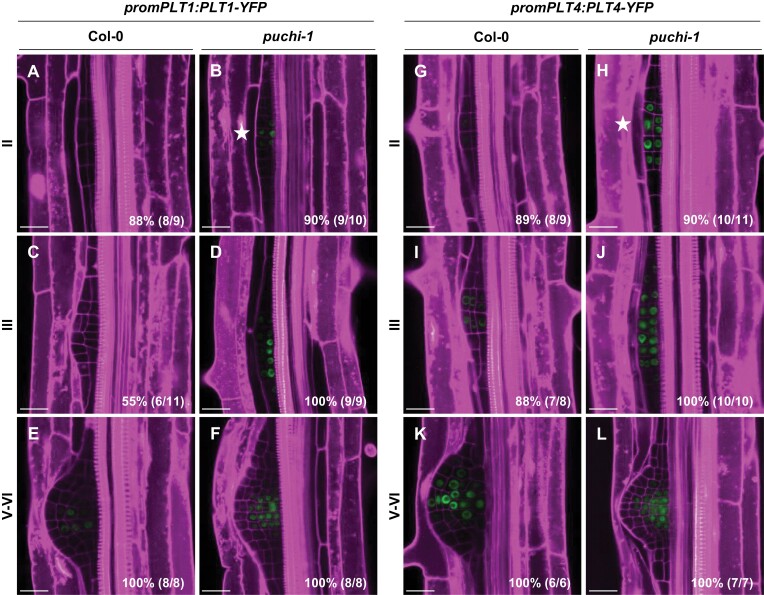
*PUCHI* loss of function results in distinct expression patterns of meristematic phase regulator *PLT* genes during LRP development. (A–F) and (G–L) Expression pattern of *promPLT1:PLT1-YFP* and *promPLT4:PLT4-YFP* (green), respectively, in *puchi-1* and Col-0 LRPs. Stars indicate earlier detected signal expression during LRP outgrowth. Percentages and numbers indicate the occurrence of the represented pattern over the total number of observations. Cell walls were stained using propidium iodide (magenta). Scale bar: 25 μm.

It was previously reported that the transcription factors PLT3, PLT5, and PLT7 control the induction of *PLT1*, *PLT2*, and *PLT4* in developing LRPs (Du and Scheres, 2017). Unexpectedly, *PLT1* and *PLT4* were overexpressed and *PLT5* and *PLT7* down-regulated in *puchi-1* mutant roots, especially in the early stages of development (Fig. 1D; Supplementary Fig. S4A). To analyse the impact of *PUCHI* loss of function on the expression pattern of root meristem-related *PLT* genes, transgenic reporter constructs *promPLT1:PLT1-YFP*, *promPLT2:PLT2-YFP*, *promPLT3:PLT3-YFP*, and *promPLT4:PLT4-YFP* were introduced into the *puchi-1* mutant background, and their expression pattern compared with that in the WT background (Galinha *et al.*, 2007; Hofhuis *et al.*, 2013). Consistent with our time-course RNAseq results, we observed earlier *PLT1-YFP* and *PLT4-YFP* expression in stages II–III (18–24 h after gravistimulation) in *puchi-1* LRPs compared with the WT (Fig. 2A–F, G–L). Conversely, *PLT3-YFP* expression was weaker in *puchi-1* LRPs, while the *PLT2–YFP* construct did not display major changes in expression in *puchi-1* compared with the WT before LRP emergence (Supplementary Fig. S4B–G, H–M). Hence, our results reveal that PUCHI inhibits expression of *PLT1* and *PLT4*, two key root meristem transcription factor genes, during the early stages of LRP formation.

### PUCHI represses expression of QC markers during the early LR development phase

**Fig. 3.**
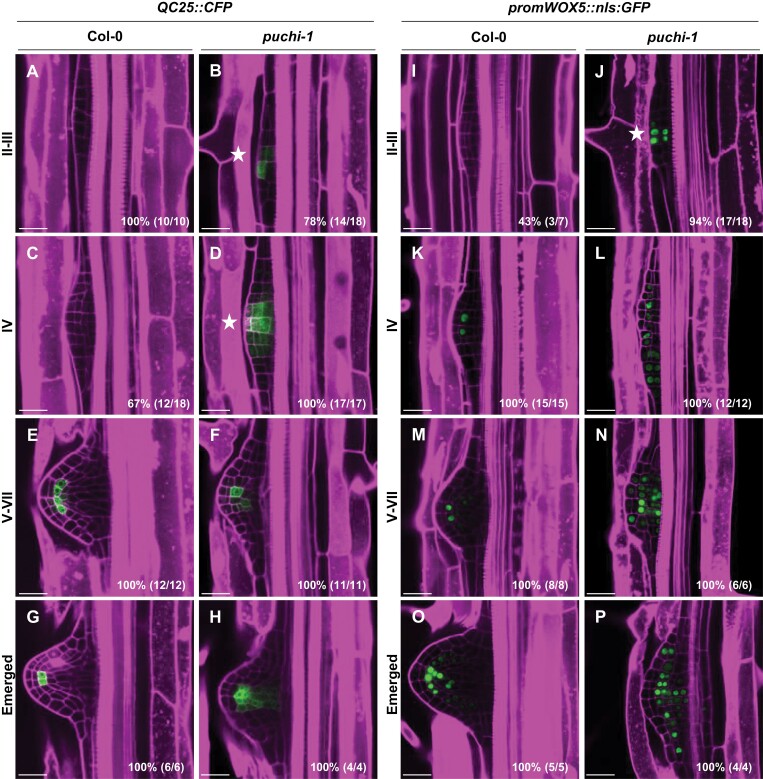
*PUCHI* loss of function impacts QC marker gene expression patterns during LRP formation. Confocal microscopy images of expression of QC reporters (A–H) *QC25::CFP* and (I–P) *promWOX5::nls:GFP* (green) in LRPs in Col-0 and *puchi-1*. Stars indicate earlier detected signal expression during LRP outgrowth. Percentages and numbers indicate the occurrence of the represented pattern over the total number of observations. Cell walls were stained using propidium iodide (magenta). Scale bar: 25 μm.

During the first phase of LR organogenesis, anticlinal, periclinal, and tangential cell divisions progressively generate a four-layer LRP that grows against the overlying endodermal layer (Malamy *et al.*, 1997; von Wangenheim *et al.*, 2016). A major developmental transition occurs at this stage, during which the LRP first expresses QC marker genes in inner central cells and acquires a dome shape as it breaks through the endodermis (Lucas *et al.*, 2013; Goh *et al.*, 2016; von Wangenheim *et al.*, 2016). The early expression of meristematic *PLT* genes suggests that the transition from the early, morphogenetic to the second, meristematic phase occurred prematurely in the *puchi-1* background. To test this hypothesis and confirm modifications in QC marker gene expression observed in the RNAseq data (Supplementary Fig. S3) (Nawy *et al.*, 2005), we investigated meristem establishment in *puchi-1* LRPs using QC marker constructs *QC25::CFP* and *promWOX5::nls:GFP* (ten Hove *et al.*, 2010; Goh *et al.*, 2016). Expression of *QC25::CFP* in Col-0 LRPs was first detected in a few central cells in the second outermost layer from stages IV–V onwards (Fig. 3C, E, G) (Goh *et al.*, 2016; Du and Scheres, 2017). In contrast, in a *puchi-1* loss-of-function background, *QC25::CFP* expression could be detected in young LRPs as early as stage II (Fig. 3B, D), and later in a wider domain compared with the WT (Fig. 3G, H). Similarly, *promWOX5::nls:GFP* was expressed prematurely in stage II in inner cell layers of *puchi-1* LRPs which correlates with *WOX5* up-regulation observed in *puchi-1* RNAseq data as compared with the WT at 18 h after LR induction (Fig. 3I, J; Supplementary Fig. S3). Furthermore, the *promWOX5::nls:GFP* expression domain stretches to inner cell layers, but also to flanking cells at later stages in *puchi-1* LRPs (Fig. 3K–P). Altogether, our observations show that expression of QC marker genes occurs earlier and in a wider domain in *puchi-1* LRPs. We conclude that PUCHI is required to delay the activation of QC marker gene expression during LRP development, possibly via repressing *PLT1* and *PLT4* expression at early stages.

### Auxin and cytokinin signalling patterns are modified in *puchi* LRPs

**Fig. 4.**
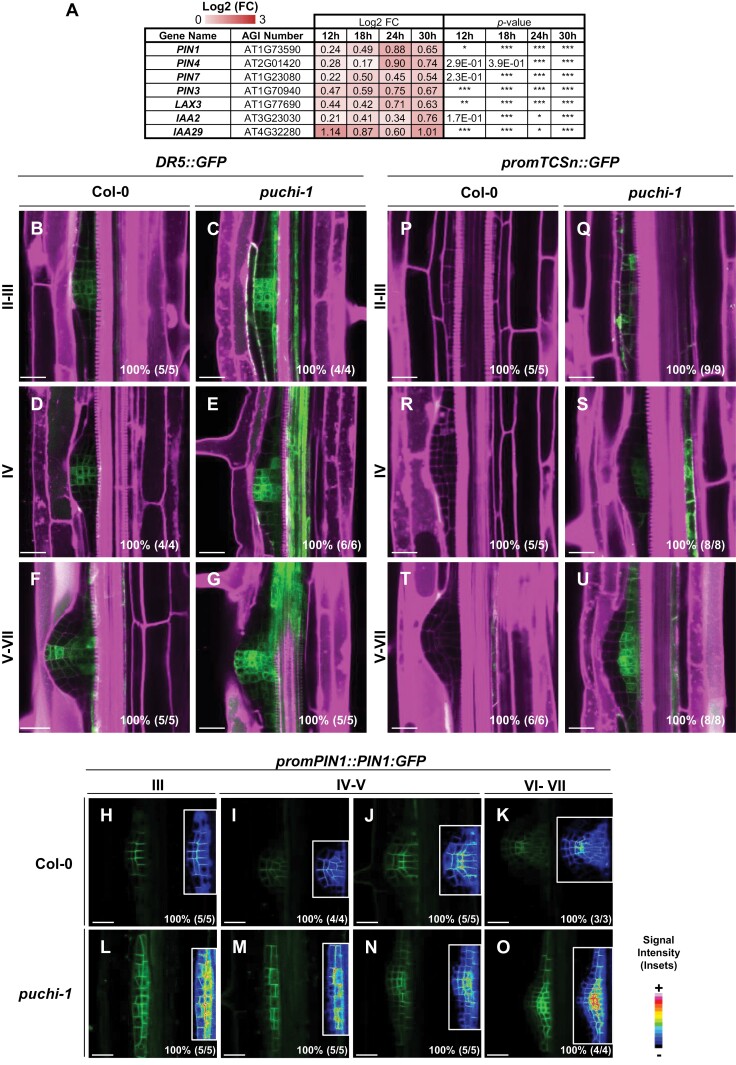
*PUCHI* loss of function alters auxin and cytokinin signal distribution in developing LRPs. (A) Heat map of selected auxin transporter gene patterns up-regulated in *puchi-1* compared with the Col-0 background during LRP formation. Log2-fold change (FC) of expression in *puchi-1* compared with the WT is given. Statistical analysis on the three independent RNAseq replicates were performed using the DESeq2 package and Wald test: *P<0.05; **P<0.02; ***P<0.01. (B–G) *DR5::GFP* (green), (H–O) *promPIN1::PIN1:GFP* (green), and (P–U) *promTCSn::GFP* (green) expression during LR development. Percentages and numbers indicate the occurrence of the represented pattern over the total number of observations. (B–G) and (P–U) Cell walls were stained using propidium iodide (magenta). (H–O) (inset) Signal intensity monitor; (blue) low intensity; (red) high intensity. Scale bar: 25 μm.

Auxin distribution regulates *PLT* gene expression and contributes to LRP organization (Laskowski *et al.*, 2008; Peret *et al.*, 2014; Mähönen *et al.*, 2014; Du and Scheres, 2017; Scheres and Krizek, 2018). Interestingly, our transcriptomic analysis revealed that genes induced by auxin, such as *IAA2*, or encoding auxin transporters, such as *PIN1*, *PIN3*, *PIN4*, and *PIN7*, were up-regulated in the early stages (18–24 hpg) of LR development in *puchi-1* (Fig. 4A; Supplementary Fig. S3) (Bishopp *et al.*, 2011). We used the *DR5::GFP* synthetic auxin response reporter to visualize the pattern of auxin signalling in *puchi-1* LRPs compared with the WT (Friml *et al.*, 2003). As previously described (Benková *et al.*, 2003), the *DR5::GFP* reporter revealed an auxin response gradient in developing WT LRPs with a maximum close to the LRP tip (Fig. 4B, D, F). In *puchi-1* mutant LRPs too, the *DR5::GFP* reporter signal was non-uniformly distributed and displayed predominant expression in the centre of the primordium in contrast to its flanks (Fig. 4C, E, G). However, in comparison with the WT situation, the *DR5::GFP* expression domain was broader and extended closer to the LRP base in *puchi-1*, especially in primordia from stage IV onwards. Thus, in contrast to a previous report based on the *DR5::GUS* expression pattern (Hirota *et al.*, 2007), our data suggest that PUCHI is required for the establishment of a WT-like auxin signal gradient during LRP development. We therefore investigated the spatial distribution of PIN1 proteins in Col-0 and *puchi-1* LRPs using a *promPIN1:PIN1-GFP* construct (Benková *et al.*, 2003). This revealed that PIN1 distribution is strongly impaired in *puchi-1* LRPs from stage III onwards, displaying a higher signal in inner cell layers compared with the WT (Fig. 4H–O).

Previous studies have shown that cytokinin impacts PIN1 polarization in LRPs from stage III onwards by reallocating PIN1 to periclinal membranes (Marhavý *et al.*, 2011, 2014). To test whether *puchi-1* LRP defects are correlated with perturbations in cytokinin signalling patterning, the *promTCSn::GFP* reporter was introduced into the *puchi-1* mutant background and its expression compared with that in WT LRPs (Zürcher *et al.*, 2013). In the WT, *promTCSn::GFP* expression was not detected in LRPs before stage VII, and from stage VII onwards the expression of this cytokinin signalling reporter was restricted to provascular cells as previously described (Fig. 4P, R, T; Bielach *et al.*, 2012). In contrast, *promTCSn::GFP* expression was detected in central cells in the inner layers and extended to flanking regions of *puchi-1* LRPs from stage II onwards (Fig. 4Q). In later stages, cytokinin signalling was observed in LRP flanks (Fig. 4S, U). We conclude that *PUCHI* loss of function compromises the establishment of auxin and cytokinin response patterns in developing LRPs.

## Discussion

**Fig. 5.**
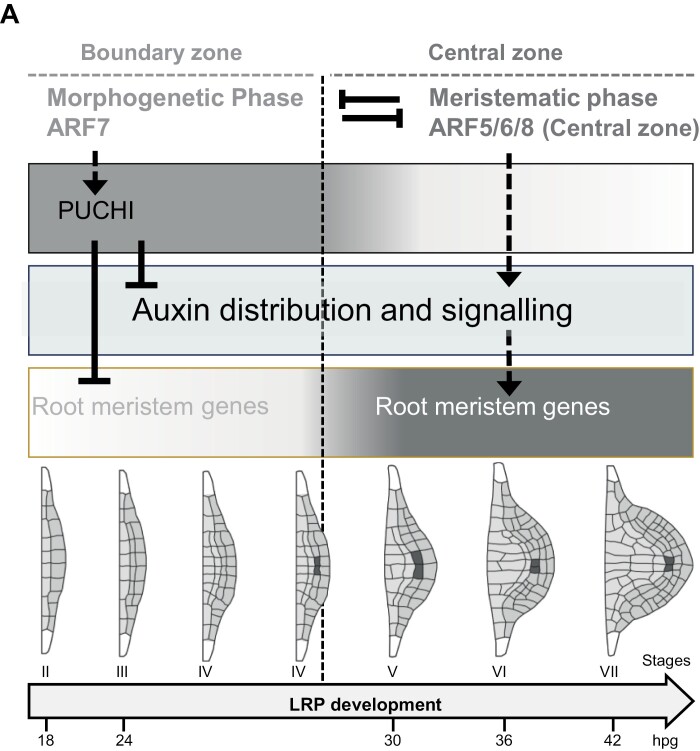
*PUCHI-*dependent early repression of meristem establishment. (A) Hypothetical model showing the role of *PUCHI* at early stages of LRP formation; *PUCHI* acts as a key regulator of spatiotemporal distribution of auxin balance and represses specific meristematic module genes.

Two successive phases were previously described during the process of LR organogenesis before the emergence of a new LR. During the first so-called morphogenetic phase, cell proliferation generates a four-cell-layered primordium that grows against the overlying endodermis layer. Only at this stage, at about mid-term of the full developmental time course of the LRP, do anatomic features reminiscent of the meristem stem cell niche become apparent in the centre of the primordium, where expression of QC marker genes initiates (Lavenus *et al.*, 2015; Voß *et al.*, 2015; Goh *et al.*, 2016). Onset of QC marker gene expression at this transition stage is dependent on the previous establishment of the layered pattern that is controlled by the SHR/SCR pathway (Goh *et al.*, 2016; von Wangenheim *et al.*, 2016). Next, during the following developmental steps, the root meristem organization in the centre of the growing primordium becomes more and more complex while surrounding cells retain LRP boundary characteristics (Malamy *et al.*, 1997; Lavenus *et al.*, 2015). After LR emergence, the QC may maintain the indeterminate growth of the LR by repressing differentiation of the surrounding meristematic cells as described in primary root meristems (van den Berg *et al.*, 1995). Many transcriptional regulators were shown to be dynamically expressed during the process of LR organogenesis and may participate in the control of cell proliferation and the acquisition of new cell identities. Remarkably, inference of the GRN controlling LR development suggested that it is organized in two genetic subnetworks whose mutual inhibition may explain LRP development patterning (Lavenus *et al.*, 2015; Voß *et al.*, 2015). One genetic subcircuit gathers genes, including those encoding the transcription factors ARF7 and PUCHI, that are expressed at early stages in the whole primordium and only in its base in later stages. The second genetic module gathers transcription factors such as ARF5 and PLT4 whose expression spans the central domain of the primordium where the meristem stem cell niche is being established, only in the second phase of its development (Fig. 5).

The transcription factor PUCHI belongs to the former genetic module and is expressed very early in the developing LRP from stage I (Hirota *et al.*, 2007; Goh *et al.*, 2019; Trinh *et al.*, 2019). Interestingly, the correct timing of *PUCHI* induction is essential: the sequential induction of *ARF7*, *LBD16*, and, only then, *PUCHI* was shown to be critical for the correct progression of LR formation as premature expression of *PUCHI* disrupted LRP initiation by repressing auxin-induced root branching (Goh *et al.*, 2019). However, how PUCHI function at early stages influences subsequent LRP development remained unclear. Here, we show that meristematic genes, *PLT1* and *PLT4*, as well as root meristem QC marker constructs, *QC25::CFP* and *promWOX5::nls:GFP*, are expressed ectopically at earlier stages and in wider domains in *puchi-1* developing LRPs as compared with the WT. Conversely *PLT5* and *PLT7*, that belong together with *PUCHI* to the group of genes expressed at early stages of LRP development (Lavenus *et al.*, 2015), are underexpressed in the *puchi-1* loss-of-function background. Although such perturbations are not observed for all the genes in each gene subnetwork, respective changes in the expression patterns of *PLT5*, *PLT7*, and root meristem genes in *puchi-1* mutant LRPs are consistent with the hypothesis that, at the level of the LR GRN system, early expressed and root meristematic genes belong to two mutually exclusive subcircuits (Lavenus *et al.*, 2015). PUCHI participates in the induction of *PLT5* and *PLT7* expression during LR formation, with *PLT5* probably being a direct target of the transcription factor PUCHI. We also show for the first time that PUCHI, which is predominantly expressed at early stages of LRP development, is required for the correct timing of meristem establishment and especially represses premature expression of key meristematic genes at early stages of LR development, namely *PLT1*, *PLT4*, and *WOX5*. Remarkably, this is clearly different from the role of PLT3, PLT5, and PLT7 transcription factors which were shown to be required for the correct progression of LRP development and its transition to the meristematic phase (Du and Scheres, 2017). These data point to a complex influence of PUCHI on the GRN controlling LR development, as its control over the expression of its direct target genes may translate into intricate effects on the gene system dynamics.

This raises the question of how PUCHI regulates cell identity acquisition in developing LRPs. Interestingly, cross analysis of three lists of PUCHI-dependent genes highlighted *CRF1* as a likely direct target gene of PUCHI. This is also supported by *in silico* predictions of PUCHI target genes based on correlated, but delayed, expression profiles (Lavenus *et al.*, 2015). *CRF1* encodes an AP2/ERF transcription factor that, redundantly with other CRFs, mediates part of the cytokinin signalling pathway modulating various plant development processes and responses to abiotic stresses, in interaction with other hormonal signalling pathways (Rashotte *et al.*, 2006). Interestingly, phenotypical analyses of multiple mutant combinations and overexpressing lines revealed a complex and redundant influence of CRFs on primary root growth and suggested that at least some members promote lateral root formation (Raines *et al.*, 2016). CRF-dependent expression of PIN family, auxin transporter genes was shown to contribute to the regulation of primary root growth and lateral root formation by cytokinins (Šimášková *et al.*, 2015). The expression pattern of the cytokinin reporter construct *promTCSn::GFP* and the distribution of the PIN1 reporter PIN1:GFP were shown to be altered in *puchi-1* mutant LRPs as compared with the WT (Fig. 4). In the RNAseq profiling of LR development, *CRF1* is the only *CRF* gene displaying such expression dynamics typical of the early expressed subnetwork and positively controlled by PUCHI (Supplementary Fig. S3). Thus, *CRF1* may represent a key target of PUCHI impacting the cytokinin- and auxin-dependent pathways that regulate LRP organogenesis and patterning.

Interestingly, a number of other transcription factors of the AP2/ERF family were identified as putative PUCHI targets supported by both the RNAseq dataset and the DAP-seq dataset (*PLT5*, *ERF2*, *ERF12*, *ERF53*, *ERF113*, and *DDF1*) (Supplementary Fig. 5B; Supplementary Table S1). Transcription factors of the AP2/ERF family are involved in the regulation of a wide range of developmental processes, in many cases in relation to hormone or stress signalling networks (Horstman *et al.*, 2014; Feng *et al.*, 2020). Interestingly, ERF12 was shown to regulate flower development, possibly by orchestrating the sequential induction and stabilization of reproductive developmental programmes in shoot apices together with PUCHI and related transcription factors (Chandler and Werr, 2020). The expression dynamics of *PLT5* during LR development previously suggested that it was indeed controlled by PUCHI (Lavenus *et al.*, 2015). Redundantly with PLT3 and PLT7, PLT5 was shown to control cell division patterning, correct auxin signal distribution, and WT progression of LRP development including the onset of root meristem gene expression such as of *PLT1*, *PLT4*, and *WOX5* (Du and Scheres, 2017). Both *PLT5* and *PLT7* expression are down-regulated in the *puchi-1* mutant background, and auxin signal distribution and PIN1 localization are also impaired (Fig. 4; Supplementary Fig. S3). Expression of *PLT3* and/or modification in expression of PLT5/7-independent genes in *puchi-1* may explain the observed differences between *plt3/5/7* and *puchi-1* LRP phenotypes.

Several genes involved in hormone homeostasis or signalling have also been identified among the PUCHI-dependent genes (Supplementary Figs S3, S5B; Supplementary Table S1). Auxin and cytokinin are key phytohormones whose roles in functional patterning of meristems and organ primordia have been well described, although in many cases the multiple feedback loops connecting GRNs and hormonal homeostasis and signalling pathways make any sequential order of molecular events difficult to establish (Chandler and Werr, 2015; Salvi *et al.*, 2020). Here, we show that auxin and cytokinin signalling patterns are impaired in *puchi-1* mutant LRPs. Interestingly, previous reports have shown higher auxin contents in the roots of 5-day-old *puchi-1* seedlings compared with the WT (Goh *et al.*, 2019). Alternatively, PUCHI may modulate auxin signalling processes and responses downstream of auxin accumulation, as suggested by the observation that ectopic expression of *PUCHI* in LR founder cells compromises the auxin signal maximum establishment without affecting auxin content (Goh *et al.*, 2019). More specifically, modification of auxin signalling distribution in *puchi-1* mutant LRPs correlates with perturbed distribution of PIN1 auxin transporters (Fig. 4A, H–O). Formation of an auxin response gradient is critical for proper LRP development (Benková *et al.*, 2003). *PLT1/2/3/4* are well-identified auxin-responsive genes that regulate the establishment and maintenance of a root stem cell niche and this, in turn, regulates the expression and localization of PIN transporters and therefore auxin distribution in the RAM and LRP (Benková *et al.*, 2003; Galinha *et al.*, 2007; Prasad *et al.*, 2011; Pinon *et al.*, 2013; Mähönen *et al.*, 2014; Du and Scheres, 2017).

Cytokinins repress the expression of the PIN auxin efflux carriers in the root meristem and in LRPs (Laplaze *et al.*, 2007; Dello Ioio *et al.*, 2008; Růžička *et al.*, 2009; Bishopp *et al.*, 2011). In addition, in the early stages of LRP development, cytokinins modulate PIN1 targeting to periclinal versus anticlinal domains of cell membranes (Marhavý *et al.*, 2011, 2014). Cytokinin signal distribution, PIN1 expression levels, and PIN1–GFP distribution are all modified in *puchi-1* LRPs. As observed for RAM initiation and maintenance, or when a RAM is regenerated after root tip excision, auxin and cytokinin signals define complementary spatial domains that may contribute to the positioning of the new stem cell niche and guide further LRP patterning (Schaller *et al.*, 2015; Efroni *et al.*, 2016). This pattern is not observed in the *puchi-1* background. Altogether, this suggests that compromised auxin and cytokinin response distribution in *puchi-1* LRPs may trigger the early ectopic expression of meristematic genes through a PIN1-regulated, auxin-dependent mechanism. Alternatively, misexpression of meristematic genes in *puchi-1* LRPs may contribute to disorganize auxin and cytokinin signal distribution and perturb LRP morphogenesis and patterning.

We recently showed that VLCFA biosynthetic enzymes are downstream targets of PUCHI in developing LRPs (Trinh *et al.*, 2019). Interestingly, an increasing body of evidence highlights crosstalk between VLCFAs and hormones in plant development (Boutté and Jaillais, 2020). For instance, VLCFAs are involved in controlling cell proliferation in shoot organs in a cytokinin-dependent, non-cell-autonomous manner (Nobusawa *et al.*, 2013; Boutté and Jaillais, 2020). Furthermore, in the VLCFA mutant *pasticcino1* (*pas1*), the abnormal polar distribution of PIN1 in specific cells results in local alteration of auxin distribution and disturbs LRP formation (Roudier *et al.*, 2010). Lastly, fused proliferating bulges generated by roots of *puchi* or VLCFA-defective mutants on auxin- and cytokinin-supplemented medium (callus-inducing medium) closely resemble those produced by roots of mutants affected in polar auxin transport in response to exogenous auxin supply, as well as those produced by cytokinin-overproducing pericycle upon treatment with a highly diffusible auxin analogue (Benková *et al.*, 2003; Geldner *et al.*, 2004; Laplaze *et al.*, 2007; Shang *et al.*, 2016; Trinh *et al.*, 2019). Altogether, these data suggest tight links between VLCFAs, cytokinin and auxin distribution, and LRP patterning. Strikingly, two distinct AP2/ERF-controlled cascades targeting VLCFA biosynthetic enzymes during LRP development were recently described, suggesting that AP2/ERF transcription factors may act as master regulators of VLCFA biosynthesis during LRP formation (Trinh *et al.*, 2019; Guyomarc’h *et al.*, 2021; Lv *et al.*, 2021). Lastly, VLCFA-containing ceramides were recently shown to participate in a feedforward loop maintaining preferential expression of the transcription factor genes *ATML1* and *PDF2*, key regulators of protodermis/epidermis differentiation, in the outer cell layer of developing LRPs (Nagata *et al.*, 2021). This function may involve direct binding of these VLCFA-containing ceramides to the START domain of the transcription factors (Nagata *et al.*, 2021). *ATML1* and *PDF2* expression dynamics are lost in *puchi-1* mutant LRPs (Supplementary Fig S7G, H). Thus, we hypothesize that PUCHI-dependent VLCFA regulation could participate actively in patterning mechanisms controlling LRP organization.

In conclusion, by analysing the impact of *PUCHI* loss of function on the progression of LRP development and functional patterning, we showed that PUCHI is required for the correct timing of meristem establishment and, in particular, represses premature expression of key root meristem genes at early stages of LRP development. These results are consistent with the model of an LR GRN organized in distinct genetic subcircuits whose sequential inhibitions or activations contribute to the transition from an early morphogenetic phase to a second, meristem establishment phase, as well as to the bifurcation between the central meristematic domain and the flanking domain in the developing organ. How VLCFA-, auxin-, or cytokinin-dependent patterning mechanisms may interact during this process downstream of PUCHI will require further investigations.

## Supplementary data

The following supplementary data are available at *JXB* online.

Fig. S1. The LRP phenotype of Col-0 and *puchi-1* LRPs during the time-course transcriptomic analysis after gravistimulation.

Fig. S2. GO term enrichment during the time-course transcriptomic analysis of LR formation in *puchi-1* compared with Col-0 upon gravistimulation.

Fig. S3. A heat map of selected gene patterns up- and down-regulated in *puchi-1* compared with the Col-0 background during the formation of LRPs.

Fig. S4. PLT gene expression analysis in *puchi-1* compared with Col-0 using qRT–PCR and confocal microscopy.

Fig. S5. Identification of the PUCHI direct target genes using available datasets.

Fig. S6. Predicted PUCHI target gene expression patterns in *puchi-1* and the Col-0 background during the formation of LRPs.

Fig. S7. Selected gene expression patterns in *puchi-1* and the Col-0 background during the formation of LRPs.

erac079_suppl_Supplementary_Figures_S1-S7

erac079_suppl_Supplementary_Table_S1

## Data Availability

All data to support the conclusions of this study are included in the main text and the supplementary data. The full RNAseq data discussed in this publication have been deposited in the NCBI’s Gene Expression Omnibus and are accessible through GEO Series accession number GSE199142 (https://www.ncbi.nlm.nih.gov/geo/query/acc.cgi?acc=GSE199142) (Edgar *et al.*, 2002).

## Abbreviations

DEG: differentially expressed gene
GR: glucocorticoid receptor
GO: Gene Ontology
GRN: gene regulatory network
hpg: hours post-gravistimulation
LR: lateral root
LRP: lateral root primordium
MS: Murashige and Skoog
NAA: 1-naphthalene acetic acid; NPA. *N*-1-naphthylphthalamic acid
QC: quiescent centre
RAM: root apical meristem
VLCFA: very long chain fatty acid
WT: wild type
