# Correction to: PUCHI represses early meristem formation in developing lateral roots of *Arabidopsis thaliana*

**DOI:** 10.1093/jxb/erad502

**Published:** 2023-12-23

This is a correction to: Kevin Bellande, Duy-Chi Trinh, Anne-Alicia Gonzalez, Emeric Dubois, Anne-Sophie Petitot, Mikaël Lucas, Antony Champion, Pascal Gantet, Laurent Laplaze, Soazig Guyomarc’h, PUCHI represses early meristem formation in developing lateral roots of *Arabidopsis thaliana*, *Journal of Experimental Botany*, Volume 73, Issue 11, 2 June 2022, Pages 3496–3510, https://doi.org/10.1093/jxb/erac079.

The originally published version of this manuscript omitted author Duy-Chi Trinh’s second affiliation of University of Science and Technology of Hanoi, Vietnam Academy of Science and Technology (VAST), Ha Noi, Vietnam.

These details have been corrected only in this correction notice to preserve the published version of record.

