# Supplementary material for: PUCHI represses early meristem formation in developing lateral roots of Arabidopsis thaliana
Source: J Exp Bot. 2022 Feb 28;73(11):3496–510. doi: 10.1093/jxb/erac079 (PMC9162184; doi:10.1093/jxb/erac079)
Supplement: erac079_suppl_Supplementary_Figures_S1-S7 [file erac079_suppl_supplementary_figures_s1-s7.pdf]

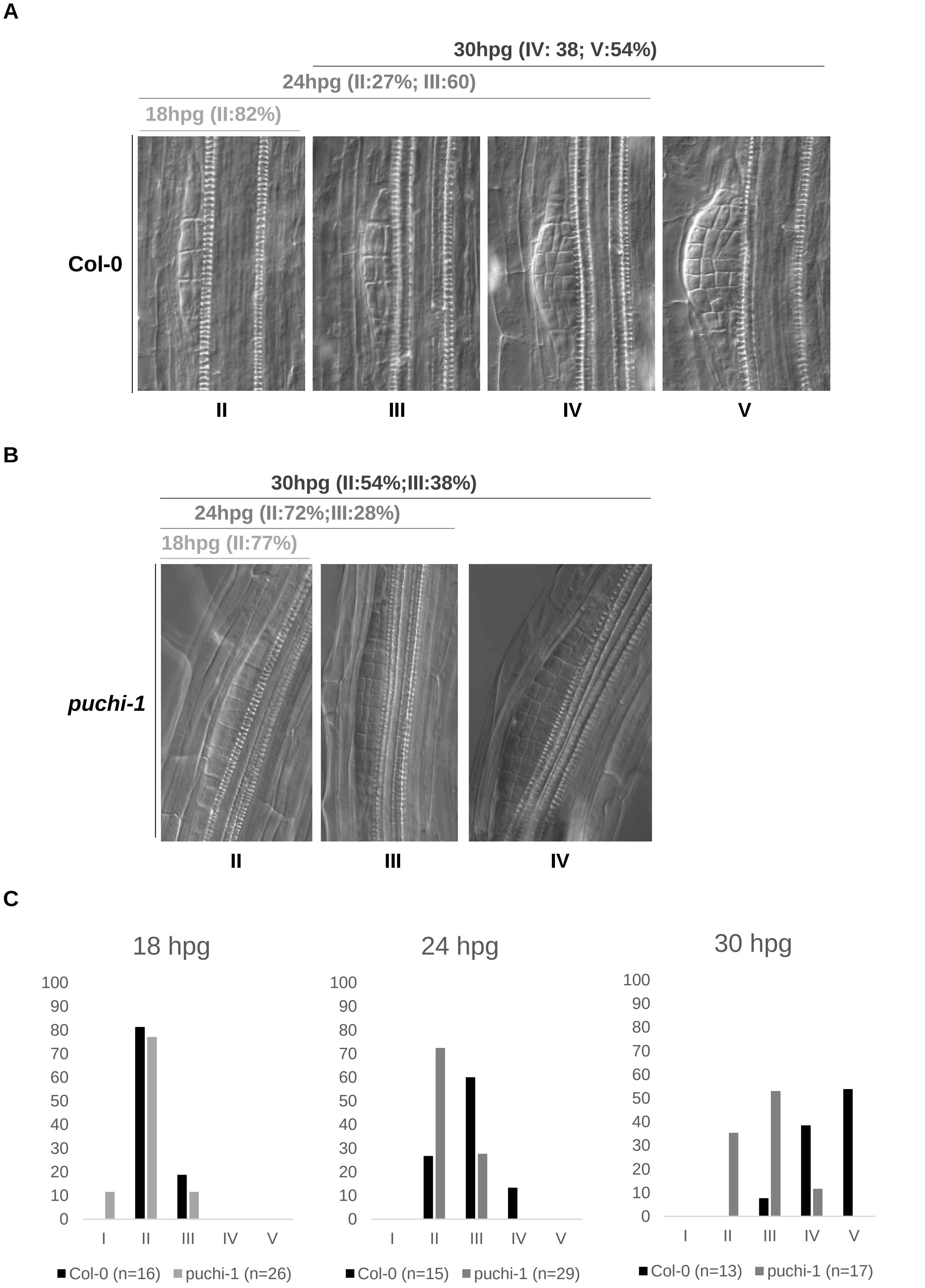

Fig. S1: **LRP phenotype of Col-0 and *puchi-1* LRP during the time-course transcriptomic analysis after gravistimulation (hpg).** (A) and (B) Brightfield images showing the LRP stages observed for each timepoint. Time points corresponds to phenotype of the LRP observed in the RNAseq samples. The percentages of occurrence for each LRP stages per time point are shown. (C) Distribution of LRP developmental stages after 18, 24, 30 hours post-gravistimulation (hpg) in Col-0 and *puchi-1* roots. Data are one biological replicate for Col-0 and two biological replicates for *puchi-1*. The number of observed seedlings is indicated (n=X).

A

GO biological process enrichment in *puchi-1* Downregulated genes						
	No LR	12h	18h	24h	30h	36h
wax biosynthetic process (GO:0010025)	0	0	36	0	27	23
cuticle development (GO:0042335)	43	0	41	13	37	22
suberin biosynthetic process (GO:0010345)	0	48	46	0	0	0
cutin biosynthetic process (GO:0010143)	0	49	0	0	0	25
fatty acid biosynthetic process (GO:0006633)	0	0	0	0	10	0
fatty acid metabolic process (GO:0006631)	0	9	0	0	0	0
very long-chain fatty acid metabolic process (GO:0000038)	0	0	25	0	19	13
lipid transport (GO:0006869)	9	0	15	5	10	7
response to fatty acid (GO:0070542)	0	0	0	5	0	0
lipid biosynthetic process (GO:0008610)	5	0	0	0	0	0
triterpenoid metabolic process (GO:0006722)	0	0	0	0	0	22
phyllome development (GO:0048827)	5	0	0	0	0	3
anther development (GO:0048653)	0	14	13	0	14	0
secondary metabolic process (GO:0019748)	0	0	0	5	0	0
response to stress (GO:0006950)	2	0	0	0	0	0
cellular response to hypoxia (GO:0071456)	0	0	0	8	0	0
response to salicylic acid (GO:0009751)	0	0	0	5	0	0
cell differentiation (GO:0030154)	0	0	0	0	0	3
GO biological process enrichment in *puchi-1* Upregulated genes						
	No LR	12h	18h	24h	30h	36h
root development (GO:0048364)	0	0	0	5	0	0
anatomical structure morphogenesis (GO:0009653)	0	0	0	4	0	0
cell growth (GO:0016049)	0	0	0	6	0	0
response to hormone (GO:0009725)	0	0	0	4	0	0
response to auxin (GO:0009733)	0	0	0	0	0	6
regulation of hormone levels (GO:0010817)	0	0	0	0	0	6
response to organic substance (GO:0010033)	4	0	0	0	0	0
response to wounding (GO:0009611)	0	0	0	0	7	0
cellular response to endogenous stimulus (GO:0071495)	0	0	0	5	0	0
response to jasmonic acid (GO:0009753)	0	0	0	0	9	0
response to fatty acid (GO:0070542)	0	0	0	0	9	0
regulation of transcription, DNA-templated (GO:0006355)	0	0	0	0	0	3

Fig. S2. GO-terms enrichment during the time-course transcriptomic analysis of LR formation in *puchi-1* compared to Col-0 upon gravistimulation. (A) Distribution of Gene Ontologies among DEGs in *puchi* was analysed using a PANTHER overrepresentation assay and Fisher test followed by a Bonferroni Correction (p*<0.05). The Heat map shows the GO terms fold enrichment of DEGs for each time point.

		Log2 FC						*p*-value					
**Gene Name**	**AGI Number**	**No LR**	**12h**	**18h**	**24h**	**30h**	**36h**	**No LR**	**12h**	**18h**	**24h**	**30h**	**36h**
**QC specific**													
**NAP**	AT1G69490	2,33	-0,06	1,08	0,14	2,98	2,02	***	9,4E-01	9,4E-02	7,9E-01	***	***
**WIP4**	AT3G20880	0,78	0,83	5,13	4,41	1,24	0,68	3,9E-01	5,6E-01	***	***	*	8,1E-02
**DUF9**	AT5G23780	0,48	0,36	1,10	0,48	0,61	0,14	3,7E-01	4,2E-01	***	1,1E-01	1,3E-01	6,4E-01
**NPY4**	AT2G23050	0,48	0,39	0,88	1,96	1,19	0,72	5,6E-01	6,1E-01	***	***	***	***
**APL4**	AT2G21590	-0,29	0,34	1,12	1,18	1,06	0,11	7,6E-01	5,4E-01	*	***	**	8,0E-01
**WOX5**	AT3G11260	0,10	0,55	0,64	-0,50	-0,75	-0,98	7,8E-01	1,1E-01	***	*	***	***
**Morphogenesis**													
**FEZ**	AT1G26870	0,18	0,39	-0,18	2,13	1,22	-0,70	8,6E-01	6,3E-01	8,0E-01	*	**	*
**SHR**	AT4G37650	0,14	0,06	0,31	0,46	0,33	0,36	5,3E-01	6,0E-01	***	***	***	***
**SCR**	AT3G54220	-0,07	0,06	-0,07	0,27	-0,06	-0,04	8,2E-01	6,5E-01	5,3E-01	6,4E-02	5,7E-01	7,3E-01
**WER/MYB66**	AT5G14750	-1,06	0,55	-0,17	0,65	-1,38	-2,44	4,3E-01	2,7E-01	6,2E-01	3,1E-01	***	***
**MIR166b**	AT3G61897	0,08	0,04	0,55	-0,18	-1,11	1,39	9,6E-01	9,7E-01	4,5E-01	8,4E-01	3,1E-01	7,4E-02
**KAN4/ATS**	AT5G42630	-1,27	0,10	-0,63	-0,98	-2,02	-1,64	**	7,6E-01	*	***	***	***
**Auxin transport**													
**LAX3**	AT1G77690	0,41	0,44	0,42	0,71	0,63	0,48	2,7E-01	**	***	***	***	***
**Auxin signalling**													
**ARF2**	AT5G62000	0,36	0,06	0,18	0,09	0,22	0,37	***	5,9E-01	1,6E-01	4,7E-01	**	***
**MP/ARF5**	AT1G19850	-0,13	-0,10	0,08	0,11	0,44	0,24	7,0E-01	6,7E-01	5,6E-01	3,2E-01	***	1,1E-01
**ARF7**	AT5G20730	-0,11	-0,07	-0,06	-0,02	-0,02	0,00	5,5E-01	5,6E-01	5,2E-01	7,6E-01	8,2E-01	9,9E-01
**ARF9**	AT4G23980	0,24	0,10	0,24	0,05	0,15	0,22	2,1E-01	3,2E-01	6,6E-02	6,3E-01	1,6E-01	6,8E-02
**ARF11**	AT2G46530	0,14	0,09	0,31	-0,07	0,20	0,18	6,2E-01	7,1E-01	2,1E-01	7,2E-01	3,4E-01	3,9E-01
**ARF19**	AT1G19220	0,13	0,14	0,17	0,35	0,17	0,27	4,8E-01	2,4E-01	8,9E-02	***	9,5E-02	*
**ETT**	AT2G33860	-0,03	0,08	0,05	-0,28	-0,28	-0,06	8,6E-01	5,7E-01	7,2E-01	4,3E-02	***	4,7E-01
**ARF4**	AT5G60450	-0,11	0,52	0,49	-0,02	0,24	0,10	3,8E-01	***	***	8,7E-01	*	4,2E-01
**ARF6**	AT1G30330	0,06	0,21	0,24	0,26	0,09	-0,07	8,7E-01	1,6E-01	*	*	3,8E-01	5,4E-01
**ARF17**	AT1G77850	0,16	0,25	0,24	-0,12	-0,06	0,05	3,7E-01	8,2E-02	8,1E-02	3,5E-01	7,3E-01	7,3E-01
**IAA11**	AT4G28640	0,09	0,14	0,22	0,25	0,43	0,58	7,5E-01	2,8E-01	1,9E-01	*	***	***
**IAA13**	AT2G33310	0,51	0,32	0,60	0,55	0,53	0,51	***	**	***	***	***	***
**IAA2**	AT3G23030	0,86	0,21	0,41	0,34	0,76	1,06	***	1,7E-01	***	*	***	***
**IAA29**	AT4G32280	1,21	1,14	0,87	0,60	1,01	1,71	1,0E-01	***	***	*	***	***
**IAA5**	AT1G15580	-0,32	0,84	1,04	1,33	0,53	1,06	6,3E-01	1,1E-01	8,7E-02	8,6E-02	3,4E-01	*
**SLR/IAA14**	AT4G14550	0,19	0,23	-0,01	0,50	0,32	-0,03	4,4E-01	1,0E-01	9,3E-01	***	*	8,9E-01
**IAA19**	AT3G15540	0,36	0,30	0,54	-0,17	0,25	0,12	5,1E-01	1,5E-01	***	1,2E-01	2,4E-01	5,4E-01
**SHY2**	AT1G04240	0,80	0,15	0,32	0,41	0,59	0,71	***	3,8E-01	6,2E-02	***	***	***
**Auxin homeostasis**													
**GH3.1**	AT2G14960	1,19	0,94	1,51	0,49	0,42	0,12	***	***	***	***	*	5,0E-01
**YDK1/GH3.2**	AT4G37390	1,53	0,47	0,77	0,55	0,59	1,17	***	*	***	1,1E-01	***	***
**GH3.3**	AT2G23170	2,32	0,61	0,88	1,09	1,60	2,08	***	4,0E-01	***	***	***	***
**GH3.5**	AT4G27260	1,24	0,85	1,17	0,87	0,80	1,09	***	***	***	***	***	***
**DAO1**	AT1G14130	0,13	-0,57	-0,87	-0,39	-0,36	-0,19	3,9E-01	***	***	**	***	1,3E-01
**VLCFA and Cuticle**													
**CDEF1**	AT4G30140	1,07	-0,71	0,43	0,20	0,49	1,19	4,6E-01	*	5,2E-01	8,2E-01	**	***
**MPK14**	AT4G36450	0,29	0,46	0,62	0,71	1,15	0,56	4,8E-01	1,8E-01	1,2E-01	*	*	2,1E-01
**ERF13**	AT2G44840	0,51	-0,57	-0,31	-1,81	0,92	0,12	3,3E-01	6,0E-01	8,3E-01	1,6E-01	1,6E-01	9,4E-01
**Signalling**													
**GLV6**	AT2G03830	-0,16	0,77	1,11	1,11	0,11	-0,72	8,5E-01	**	***	***	7,6E-01	***
**MAKR4**	AT2G39370	0,88	0,64	0,90	1,20	1,00	1,99	1,5E-01	***	***	***	***	***
**MKK4**	AT1G51660	-0,03	-0,27	-0,08	-0,52	-0,04	0,06	9,4E-01	2,3E-01	8,0E-01	*	8,0E-01	8,2E-01
**MKK5**	AT3G21220	-0,37	-0,19	-0,21	-0,34	-0,01	-0,31	7,6E-02	2,7E-01	1,4E-01	***	9,7E-01	***
**MPK3**	AT3G45640	0,32	-0,56	-0,25	-0,99	0,51	0,06	5,4E-01	3,7E-01	7,3E-01	***	8,9E-02	9,2E-01
**TOLS2**	AT4G37295	0,32	0,56	0,67	0,34	0,22	0,55	1,9E-01	**	***	*	1,5E-01	***
**Transcription factors**													
**FLP/MYB124**	AT1G14350	-0,65	-0,77	-0,68	-0,71	-0,39	-0,54	1,5E-01	***	***	***	4,3E-02	***
**GATA23**	AT5G26930	1,08	1,55	1,79	2,80	2,38	3,57	4,5E-01	***	***	***	***	***
**LBD16**	AT2G42430	-0,09	-0,93	-0,89	-1,11	-0,86	-0,44	8,3E-01	***	***	***	***	***
**LBD18**	AT2G45420	0,09	0,27	0,59	0,20	0,42	0,20	8,5E-01	1,5E-01	***	3,5E-01	*	4,8E-01
**LBD29**	AT3G58190	0,94	0,39	0,62	0,82	0,99	1,13	***	1,7E-01	***	***	***	***
**Cytokinins**													
**CRF1**	AT4G11140	-1,68	-2,64	-2,88	-2,40	-2,50	-2,24	***	***	***	***	***	***
**CRF3**	AT5G53290	0,09	0,58	0,71	0,54	0,65	0,38	7,0E-01	***	***	***	***	***
**CRF2**	AT4G23750	0,56	0,48	0,67	0,40	0,16	-0,18	1,9E-01	5,7E-02	***	***	2,5E-01	2,8E-01
**CRF4**	AT4G27950	-0,51	0,29	0,22	0,66	0,38	-0,21	2,4E-01	4,9E-01	4,2E-01	*	7,7E-02	5,4E-01
**APT5**	AT5G11160	-1,05	0,10	0,23	-0,14	-0,14	-0,52	1,4E-01	9,1E-01	6,3E-01	6,3E-01	6,1E-01	6,9E-02
**AHP6**	AT1G80100	-1,59	-1,24	-1,02	-0,87	-0,37	-1,56	*	5,6E-02	*	7,9E-02	4,5E-01	***
**ARR5**	AT3G48100	-0,73	-0,53	-0,12	0,10	0,02	-0,85	***	8,3E-02	3,7E-01	5,2E-01	9,5E-01	***
**ARR14**	AT2G01760	-0,04	-0,51	0,18	0,09	0,38	0,03	9,6E-01	3,0E-01	7,5E-01	8,0E-01	2,5E-01	8,9E-01
**ARR18**	AT5G58080	1,38	N/A	N/A	-0,46	-0,04	N/A	2,7E-01	N/A	N/A	7,1E-01	9,8E-01	N/A
**ARR15**	AT1G74890	-2,12	0,42	0,58	1,15	-0,99	-0,45		4,3E-01	2,1E-01	1,6E-02	1,9E-01	7,0E-01
**ARR7**	AT1G19050	-0,15	-0,07	-0,07	-0,13	-0,15	-0,67	8,6E-01	7,5E-01	7,2E-01	4,4E-01	4,4E-01	***
**IPT7**	AT3G23630	-0,60	-0,45	-0,50	-0,64	-0,56	-0,61	1,7E-01	*	***	***	***	**
**IPT1**	AT1G68460	-2,00	0,62	-1,47	0,40	-1,01	-2,40	2,4E-01	6,8E-01	2,3E-01	7,4E-01	4,4E-01	*
**IPT3**	AT3G63110	-0,62	0,06	-0,06	-0,34	-0,42	-0,27	6,7E-02	7,3E-01	7,0E-01	1,0E-01	**	2,7E-01
**CKX1**	AT2G41510	0,24	-0,24	0,01	0,89	0,82	1,31	6,8E-01	4,3E-01	9,6E-01	***	***	***
**CKX6**	AT3G63440	0,72	0,94	1,23	1,21	1,21	1,18	***	***	***	***	***	***
**CKX5**	AT1G75450	-0,71	0,12	0,16	-0,26	-0,09	-0,47	**	6,7E-01	2,5E-01	1,7E-01	7,1E-01	**
**CKX2**	AT2G19500	-0,37	N/A	N/A	N/A	-1,75	-1,77	8,7E-01	N/A	N/A	N/A	4,0E-01	1,2E-01
**LOG3**	AT2G37210	1,32	0,29	1,80	1,42	1,74	0,86	*	4,9E-01	***	***	***	***

Module 1 (Lavenus *et al*., 2015)

Module 2 (Lavenus *et al*., 2015)

Figure S3. Time-course transcriptomic analysis in Col-0 and *puchi-1* after gravistimulation. Heat map of selected gene patterns up- and down-regulated in *puchi-1* compared to Col-0 background during the formation of LRP. Statistical analysis on three independent RNAseq replicates were performed using the DESeq2 package and Wald Test: p-value: *<0.05; **<0.02; ***<0.01. Colour code for the heatmap is red for upregulated genes and blue for downregulated genes. Colour code for gene names and AGI numbers indicates the module classification (Orange: module1, Morphogenetic phase and Purple: module 2, Meristematic phase) as described in Lavenus *et al*., 2015.

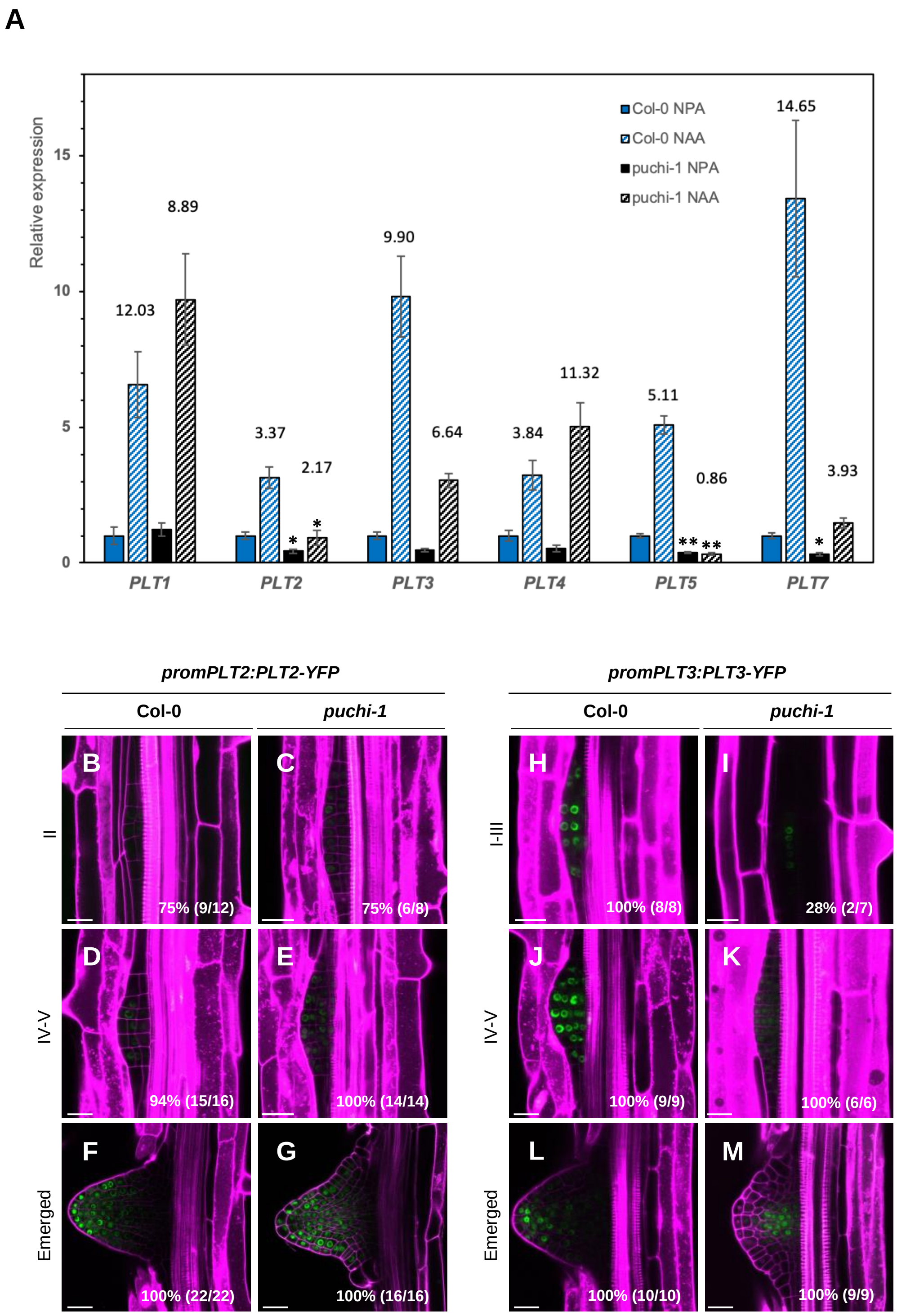

Fig. S4. Time-course transcriptomic analysis in Col-0 and *puchi-1* upon gravistimulation. (A) RT-qPCR analysis of *PLT1, PLT2*, *PLT3, PLT4*, *PLT5* and *PLT7* gene expression levels 24h after lateral root induction NPA/NAA treatment (Himanen *et al.,* 2002) in *puchi-1* and Col-0. Data are represented as mean ± SEM (standard error of the means), n=3 independent biological replicates. Significance was determined using a Student's t test (* $p< 0.05$, ** $p < 0.01$, *** $p < 0.001$). Expression pattern of (B-G) *promPLT2:PLT2-YFP* and (H-M) *promPLT3:PLT3-YFP* (green) in *puchi-1* and Col-0 LR primordia. Percentages and numbers indicate the occurrence of the represented pattern over the total number of observations. (B-M) Cell membranes were stained using propidium iodide (magenta). Scale bar: 25 µm.

A

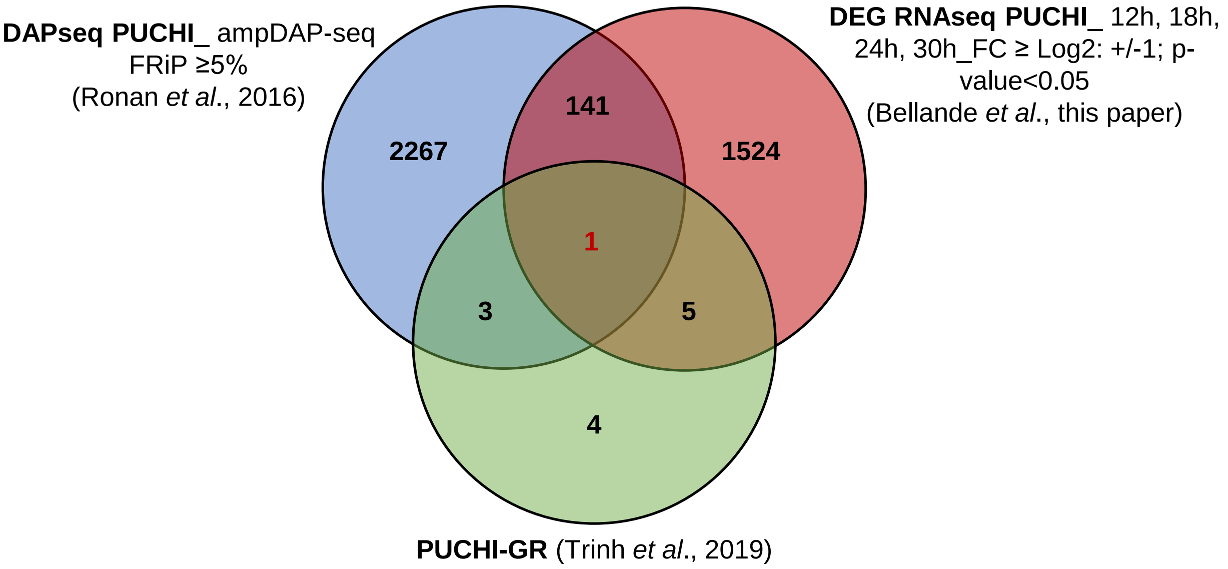

B

AGI number	TAIR Aliases	TAIR Summary
AT4G11140	CRF1	Encodes a member of the ERF (ethylene response factor) subfamily B-5 of ERF/AP2 transcription factor family. The protein contains one AP2 domain. There are 7 members in this subfamily. CRF proteins relocalize to the nucleus in response to cytokinin.
AT3G51060	SRS1, STY1	A member of SHI gene family. Arabidopsis thaliana has ten members that encode proteins with a RING finger-like zinc finger motif. Despite being highly divergent in sequence, many of the SHI-related genes are partially redundant in function and synergistically promote gynoecium, stamen and leaf development in Arabidopsis. STY1/STY2 double mutants showed defective style, stigma as well as serrated leaves. Binds to the promoter of YUC4 and YUC8 (binding site ACTCTAC)
AT1G19790	SRS7	A member of SHI gene family. Arabidopsis thaliana has ten members that encode proteins with a RING finger-like zinc finger motif. Despite being highly divergent in sequence, many of the SHI-related genes are partially redundant in function and synergistically promote gynoecium, stamen and leaf development in Arabidopsis.
AT2G28500	LBD11	
AT3G56400	WRKY70	Member of WRKY Transcription Factor
AT4G29230	NAC075	NAC domain protein involved in negative regulation of flowering.
AT5G53950	ANAC098, CUC2	Transcriptional activator of the NAC gene family, with CUC1 redundantly required for embryonic apical meristem formation, cotyledon separation and expression of STM. Proper timing of CUC2 expression is required to maintain the phyllotactic pattern initiated in the meristem. CUC2 expression in leaf sinus region is required for serration and the extent of serration is modulated by mir164A mediated repression of CUC2. Together with CUC3-DA1-UBP15 part of a regulatory module which controls the initiation of axillary meristems, thereby determining plant architecture. Regulates the axillary meristem initiation, directly binding to the DA1 promoter.
AT1G28360	ERF12	encodes a member of the ERF (ethylene response factor) subfamily B-1 of ERF/AP2 transcription factor family (ERF12). The protein contains one AP2 domain. There are 15 members in this subfamily including ATERF-3, ATERF-4, ATERF-7, and leafy petiole. Regulates floral development.
AT1G12610	DDF1	Encodes a member of the DREB subfamily A-1 of ERF/AP2 transcription factor family (DDF1). The protein contains one AP2 domain. There are six members in this subfamily, including CBF1, CBF2, and CBF3. Overexpression of this gene results in delayed flowering and dwarfism, reduction of gibberellic acid biosynthesis, and increased tolerance to high levels of salt. This gene is expressed in all tissues examined, but most abundantly expressed in upper stems. Overexpression of this gene is also correlated with increased expression of GA biosynthetic genes and RD29A (a cold and drought responsive gene). Under salt stress it induces the expression of GAOX7, which encodes ad C20-GA inhibitor.
AT2G20880	ERF53	Encodes ERF53, a drought-induced transcription factor. Belongs to the AP2/ERF superfamily, and has a highly conserved AP2 domain. Regulates drought-responsive gene expressions by binding to the GCC box and/or dehydration-responsive element (DRE) in the promoter of downstream genes. Overexpression of AtERF53 driven by the CaMV35S promoter resulted in an unstable drought-tolerant phenotype in T2 transgenic plants. Involved in heat shock response.
AT5G24110	WRKY30	member of WRKY Transcription Factor
AT5G47220	ERF2	Encodes a member of the ERF (ethylene response factor) subfamily B-3 of ERF/AP2 transcription factor family (ATERF-2). The protein contains one AP2 domain. Functions as activator of GCC boxâ€“dependent transcription. Positive regulator of JA-responsive defense genes and resistance to F. oxysporum and enhances JA inhibition of root elongation.
AT1G29670	GDSL1	GDSL-motif esterase/acyltransferase/lipase. Enzyme group with broad substrate specificity that may catalyze acyltransfer or hydrolase reactions with lipid and non-lipid substrates. The mRNA is cell-to-cell mobile.
AT2G38110	GPAT6	bifunctional sn-glycerol-3-phosphate 2-O-acyltransferase/phosphatase. Involved in cutin assembly.
AT3G16370		GDSL-motif esterase/acyltransferase/lipase. Enzyme group with broad substrate specificity that may catalyze acyltransfer or hydrolase reactions with lipid and non-lipid substrates. The mRNA is cell-to-cell mobile.
AT5G56970	CKX3	It encodes a protein whose sequence is similar to cytokinin oxidase/dehydrogenase, which catalyzes the degradation of cytokinins.
AT4G26200	ACCS7, ACS7,	Member of a family of proteins in Arabidopsis that encode 1-Amino-cyclopropane-1-carboxylate synthase, an enzyme involved in ethylene biosynthesis. Not expressed in response to IAA.
AT1G51500	ABCG12, CER5	Encodes an ABC transporter involved in cuticular wax biosynthesis. Lines carrying recessive mutations in this locus have weakly glaucous stem surface, and relative elevated secondary alcohols and ketones.
AT1G43160	ERF113, RAP2.6	encodes a member of the ERF (ethylene response factor) subfamily B-4 of ERF/AP2 transcription factor family (RAP2.6). The protein contains one AP2 domain. There are 7 members in this subfamily.
AT5G57390	AIL5, PLT5	Encodes a member of the AP2 family of transcriptional regulators. May be involved in germination and seedling growth. Mutants are resistant to ABA analogs and are resistant to high nitrogen concentrations.essential for the developmental transition between the embryonic and vegetative phases in plants. Overexpression results in the formation of somatic embryos on cotyledons. It is also required to maintain high levels of PIN1 expression at the periphery of the meristem and modulate local auxin production in the central region of the SAM which underlies phyllotactic transitions. Acts redundantly with PLT3 and 7 in lateral root pattern formation.
AT2G14960	GH3.1	encodes a protein similar to IAA-amido synthases. Lines carrying an insertion in this gene are hypersensitive to auxin.
AT1G24470	KCR2	Encodes one of the two Arabidopsis homologues to YBR159w encoding a S. cerevisiae beta-ketoacyl reductase (KCR), which catalyzes the first reduction during VLCFA (very long chain fatty acids, >18 carbon) elongation: KCR1 (At1g67730), KCR2 (At1g24470). Complementation of the yeast ybr159Delta mutant demonstrated that the two KCR proteins are divergent and that only AtKCR1 can restore heterologous elongase activity similar to the native yeast KCR gene.
AT1G46264	HSFB4, SCZ	Encodes SCHIZORIZA, a member of Heat Shock Transcription Factor (Hsf) family. Functions as a nuclear factor regulating asymmetry of stem cell divisions.
AT1G71030	MYBL2	Encodes a putative myb family transcription factor. In contrast to most other myb-like proteins its myb domain consists of a single repeat. A proline-rich region potentially involved in transactivation is found in the C-terminal part of the protein. Its transcript accumulates mainly in leaves.
AT4G36260	SRS2, STY2	A member of SHI gene family. Arabidopsis thaliana has ten members that encode proteins with a RING finger-like zinc finger motif. Despite being highly divergent in sequence, many of the SHI-related genes are partially redundant in function and synergistically promote gynoecium, stamen and leaf development in Arabidopsis. Encodes protein with a single zinc finger motif and a members of a small gene family of putative transcription factors in which the SHORT INTERNODES (SHI) gene is found. STY2/STY1 double mutants showed defective style, stigma as well as serrated leaves.

Figure S5. Identified PUCHI target genes in different datasets. (A) Venn diagram showing the number of targeted genes by PUCHI using DAPseq (Ronan *et al*., 2016; http://neomorph.salk.edu/dap_web/pages/index.php), RNAseq analysis in *puchi-1* background after gravistimulation (This paper) and PUCHI-GR inducible system (Trinh *et al*., 2019). Genes in pPUCHI::PUCHI:GR in *puchi-1* seedlings showed an upregulation (fold change ≥ 1.5, p-value ≤0.05 as determined by Welch two sample t-test) in the following conditions: NAA CHX DEX vs NAA CHX. (GR: GLUCOCORTICOID RECEPTOR; DEX: dexamethasone; CHX: cycloheximide). (B) The common gene (red) between the three datasets and 25 genes (Auxin-; cytokinin-; VLCFA-; LR-; meristematic- related and transcription factors) that were common between two datasets (among the 150 genes, see suppl. Table 1 for detailis) were selected and their AGI number, aliases and TAIR summary are shown in the table.

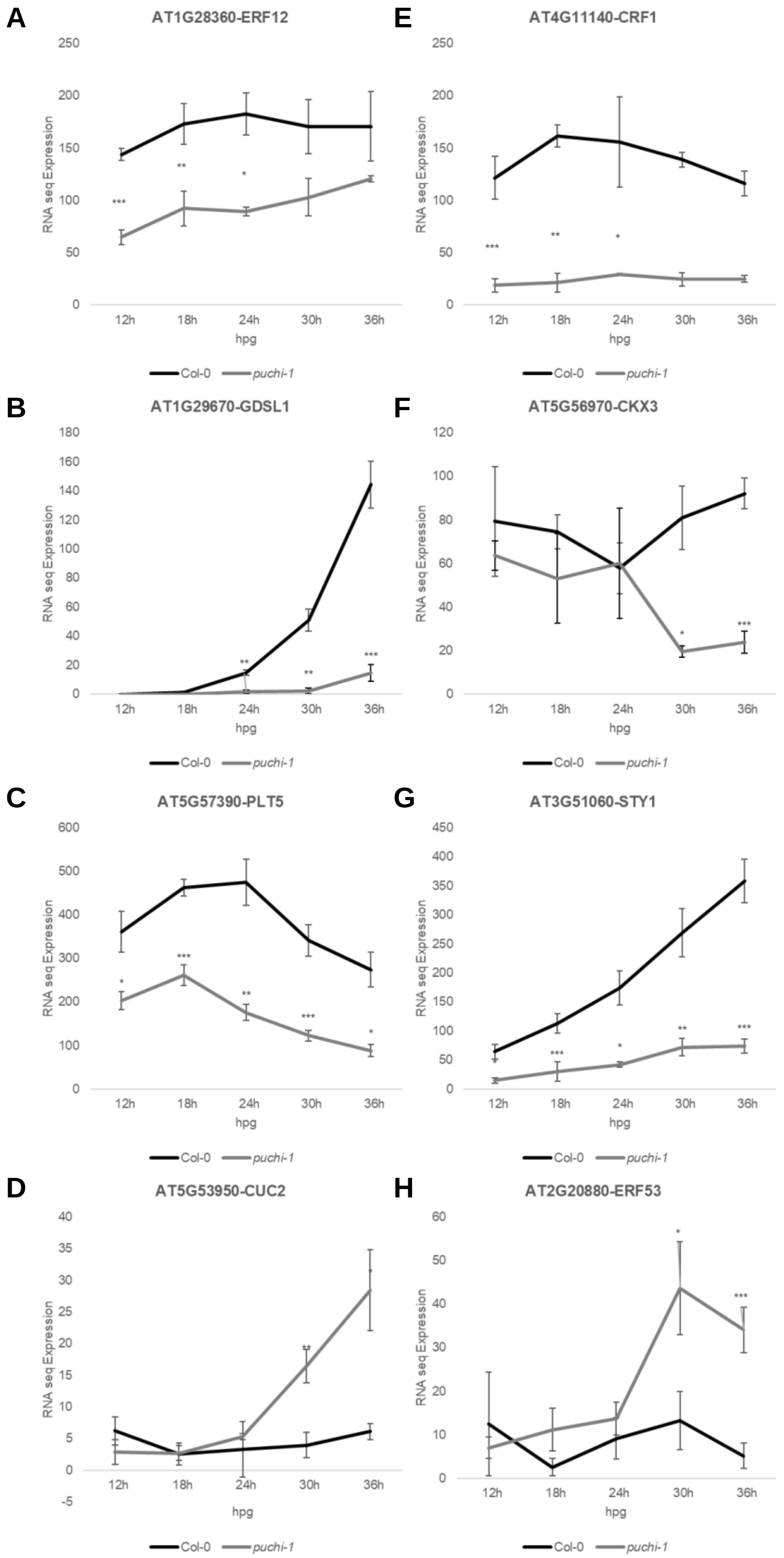

Figure S6. Expression patterns of the 8 predicted PUCHI target genes. (A-H) Gene expression patterns in *puchi-1* and Col-0 background during the formation of LRP. Data are means +/- SE of three replicates per time point from the transcriptomic dataset. Statistical analysis on RNAseq replicates were performed using DESeq2 package and Wald Test: p-value: *<0.05; **<0.02; ***<0.01.

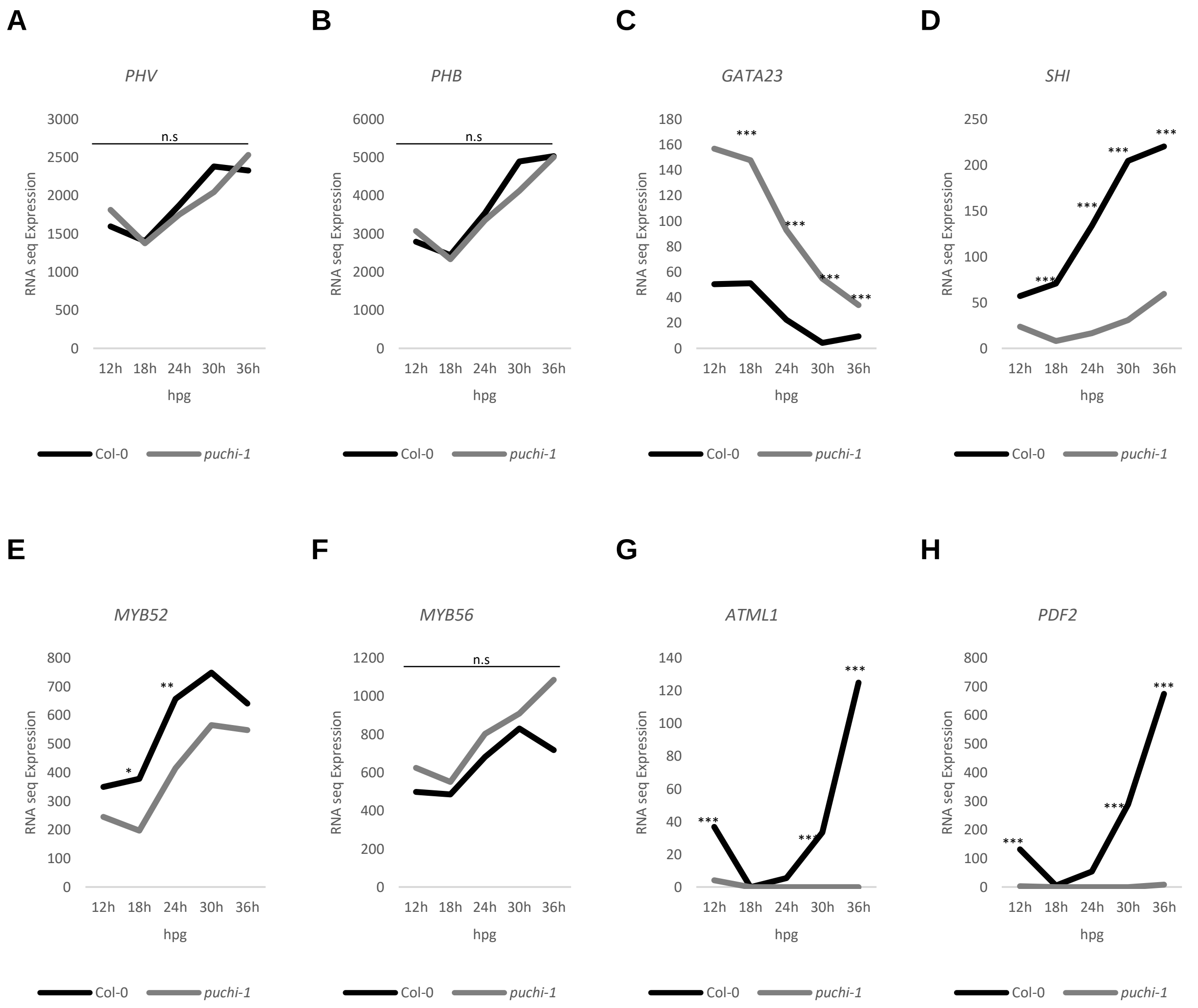

Fig. S7: **Selected gene expression analysis in Col-0 and *puchi-1* after gravistimulation. (A-H)** Gene expression patterns in *puchi-1* and Col-0 background during the formation of LRP. Data are means +/- SE of three replicates per time point from the transcriptomic dataset. Statistical analysis on RNAseq replicates were performed using DESeq2 package and Wald Test: p-value: *<0.05; **<0.02; ***<0.01.
